# A harmonized and spatially explicit dataset from 16 million payments from the European Union's Common Agricultural Policy for 2015

**DOI:** 10.1016/j.patter.2021.100236

**Published:** 2021-04-09

**Authors:** Kimberly A. Nicholas, Frida Villemoes, Edmund Aristid Lehsten, Mark V. Brady, Murray W. Scown

**Affiliations:** 1 Lund University Centre for Sustainability Studies (LUCSUS), Box 170, 22100 Lund, Sweden; 2 Agrifood Economics Centre, Department of Economics, Swedish University of Agricultural Sciences (SLU), 22070 Lund, Sweden; 3 Centre for Environmental and Climate Science (CEC), Lund University, 22362 Lund, Sweden; 4 Copernicus Institute of Sustainable Development, Utrecht University, 3584 CB Utrecht, the Netherlands

**Keywords:** agricultural subsidies, public spending, transparency, rural development, income support, farm payments, sustainable agriculture, income inequality, greenhouse gas emissions, biodiversity

## Abstract

The Common Agricultural Policy (CAP) is the largest budget item in the European Union, but varied data reporting hampers holistic analysis. Here we have assembled the first dataset to our knowledge to report individual CAP payments by standardized CAP funding measures and geolocation. We created this dataset by translating, geolocating to the county or province (NUTS3) level, and consistently harmonizing payment measures for over 16 million payments from 2015, originally reported by EU member states and compiled by the Open Knowledge Foundation Germany. This dataset and code allow in-depth analysis of over €60 billion in public spending by purpose and location for the first time, which enables both individual payment tracing and analysis by aggregation. These data are representative of the distribution of annual CAP payments from 2014 to 2020 and are of interest to researchers, policy makers, non-governmental organizations, and journalists for evaluating the distribution and impacts of CAP spending.

## Highlights

• New dataset of €61 billion in public spending under the EU's largest budget item
• Purpose and location mapped from 16 million individual payments in Europe from 2015
• Enables both detailed and aggregated analysis of agricultural spending
• Dataset well validated and can be combined with outcome data to assess policy effect

## The bigger picture

Transparency is a key principle of the European Union (EU), but previous spending on the EU's largest budget item, farm payments under the Common Agricultural Policy, has been difficult to analyze and compare at the EU level. We have created the first dataset that makes it possible to map and analyze farm payment spending by location and purpose across all member states. We hope that these data will be used to assess the effectiveness of the Common Agricultural Policy, to highlight areas where public spending is most supporting public benefits, as well as areas where spending could be redirected to more effectively support the stated goals of the policy. Sustainable agriculture is essential to meet human needs while meeting social goals, including European Commission climate and biodiversity targets. Our dataset can help to evaluate the role agricultural policy is currently playing and identify key areas where public support is needed to achieve these goals.

## Graphical abstract

**Figure:**
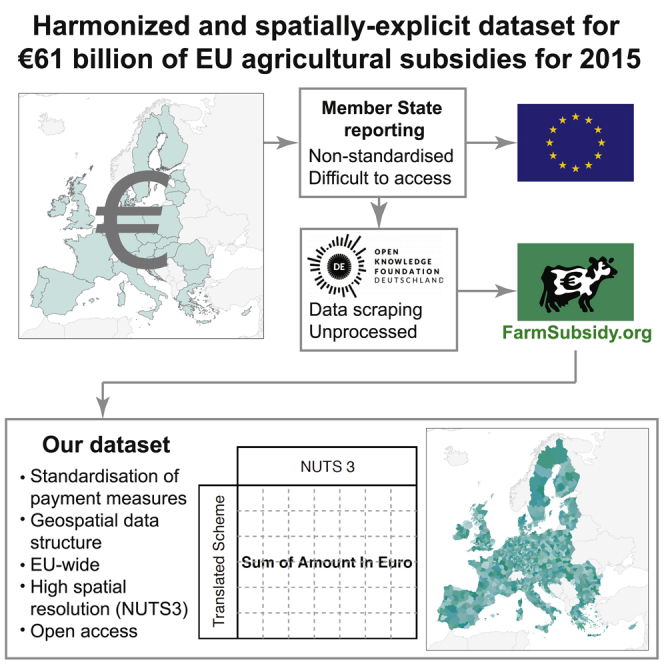

## Teaser

Public spending under the European Union's Common Agricultural Policy has been difficult to analyze at a fine spatial resolution. Here we present a dataset that for the first time harmonizes spending across member states by location and purpose drawn from 16 million individual payments in 2015. When coupled with existing data on social and environmental outcomes, our spending data enable analysis of how effectively public money is being spent to achieve policy goals.

## Introduction

Agriculture provides essential food and livelihoods for people, but land-use change, primarily driven by agriculture, also causes the majority of global biodiversity loss^1^ and 23% of climate heating.^2^ The current food system is criticized for harming both planetary and personal health, recognizing the urgent need to transform to healthy and sustainable food systems.^3^^,^^4^ Agricultural subsidies globally total over $700 billion (€640 billion),^5^ with many reinforcing harmful practices.

The European Union (EU) has pledged to be a global leader in sustainable agriculture, including making the “farm to fork” sustainable agriculture strategy a cornerstone of the European Green Deal.^6^ Currently the principal policy for European agriculture is the Common Agricultural Policy (CAP), the largest budget item in the EU. The CAP consists of two pillars: Pillar I comprises about 71% of CAP spending as direct payments to farmers and 4% to market measures, with the remaining 25% of funding supporting Pillar II programs in rural development and environmental measures.^7^

The overriding aims of the CAP are to support farmers' income, improve agricultural productivity and competitiveness, ensure a stable supply of affordable food, and support rural development, climate action, and sustainable resource management.^8^^,^^9^

However, the CAP has faced wide-ranging criticism, including for increasing income inequalities and for underresourcing goals for rural development and environmental protection by overfinancing ineffective income support.^10^^,^^11^ The CAP is currently under reform for 2021–2027.^12^ The European Commission has communicated that the future CAP should evolve in line with the Sustainable Development Goals.^13^

EU member states are obligated to report spending to comply with the EU's principle of transparency, including regulations with specific obligations for publishing CAP payment recipients.^8^ Specifically, in Article 111 of Regulation (EU) No. 1306/2013,^14^ member states are required to report the following information on a single website for at least 2 years following publication: payment beneficiaries (first and last names of individuals or full legal name of associations or companies), the municipality where the beneficiary is registered (and postal code “where available”), the amounts of payment corresponding to each measure, and “the nature and description of the measures” for both EU and member state contributions. The European Commission maintains a web page^15^ with links to each country's CAP payments reporting website, where they state, “To ensure full transparency, EU countries publish information relating to the beneficiaries of all common agricultural policy (CAP) payments on their national websites” (see supplemental information). Currently, all farms or farmers have an individual ID number, but the system depends on the individual member state, and there is no common system in the EU (R. Hießerich, Federal Ministry of Food and Agriculture Germany, personal communication, May 8, 2020).

In practice, it is currently extremely difficult to get an overview of CAP spending at a finer level than the national summaries published by member states or aggregated EU analyses published by the European Commission, because data are fragmented and incomplete. Each member state maintains its own database for reporting CAP spending, each of which uses a different format and includes different information. Data access is a problem; most of these transparency portals allow only specific searches (it is not possible to see or download all the data without writing your own code to do so), and most portals remove data older than the latest 2 years.

Crucially, there is no universal standard for the “nature and description of measures” that member states are required to report, so there has been no way to harmonize the data (by which we mean standardize payments so that their purpose, recipient, and location can be compared and aggregated between member states). Such harmonization is needed to gain a comprehensive overview of where CAP spending went and for what purpose, as well as to combine the CAP data with other datasets, for example, on environmental and social outcomes that the CAP is intended to promote, to assess the policy's effectiveness in practice.

The goal of the present study is to develop and present the first spatially explicit database of CAP spending, harmonized across measures and member states for the fiscal year 2015. To do so, we created a “Rosetta Stone” to align measure names reported between countries (called “scheme” in the raw data and our code) to a standardized list. This spending averages €58.2 billion annually over the 2014–2020 program period.^7^ The raw payments data were originally reported by EU member states, and scraped from 27 different reporting websites by the Open Knowledge Foundation Germany. They average over 600,000 records each (range: 8,600 records reported for Malta to 3,235,524 records reported for Romania), where each line represents a payout amount to a given recipient under a given measure. We performed language translation and aligned a given scheme name with the purpose of the measure by using machine translation and native speakers, consultation with local agricultural experts, and extensive data formatting and processing. The resulting database enables analysis of the purpose and location of CAP spending for the first time and facilitates future analysis of the social and environmental benefits of this spending, for example, in relation to CAP and sustainability goals.

## Results

### Creating the harmonized payments database

**Figure 1:**
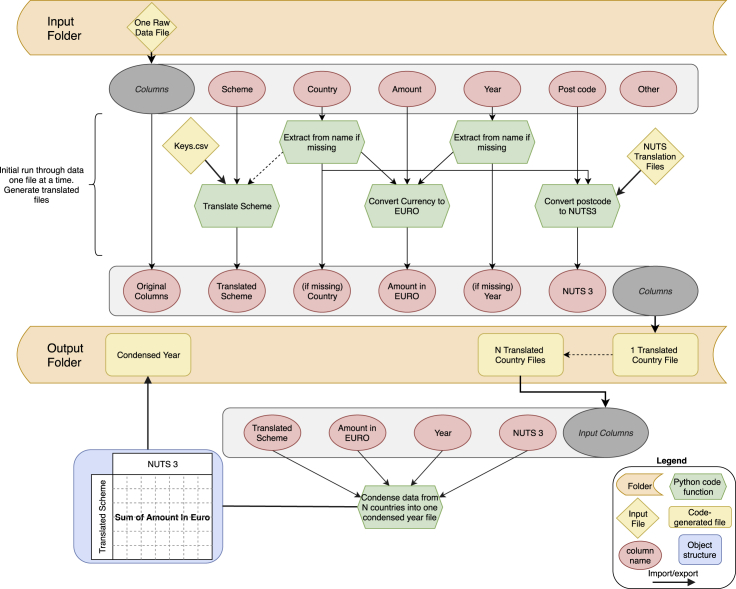
Workflow for generating the data for CAP spending by NUTS3 region within the EU and one of 102 CAP payment measures Input files include raw data from farmsubsidy.org; the keys-csv translation file to align country-specific measure names with standardized measure numbers, generated by the research team (this is the “Rosetta Stone” file, Table S2, with meta-data about measure names removed); and files to translate postal codes to NUTS regions (downloaded from EU). Steps in the Python code are shown in green. The first phase takes in raw country data from farmsubsidy.org and outputs the translated file, where rows are matched to NUTS3 regions and CAP measures. The second phase extracts the relevant columns from each country file, and for all countries within 1 year produces a condensed file of CAP spending by measure per NUTS3. See code and readme file on our GitHub.

In brief, the workflow proceeded in two stages using a Python script (Figure 1). First, we processed raw data files reporting CAP payments for each country from 2015 (obtained from farmsubsidy.org) to create a “translated” version of the country file. The translated version included additional columns appended to assign each row to a standardized measure name by using the Rosetta Stone we created as a lookup table, amount of spending in euros, and a NUTS3 region (in the EU's Nomenclature of Territorial Units for Statistical Analysis) for spatial analysis. These translated data files are suitable for individual country analysis or detailed analysis of particular measures across the EU. Second, we aggregated all of the translated country files to produce the “condensed year” file, which contains the total amount of spending for each CAP measure and NUTS3 region in the EU, suitable for broader-scale analyses and aggregation. Code to reproduce the full dataset is available on GitHub.

We used raw CAP payment data scraped from country websites by FarmSubsidy.org, which is a project of the Open Knowledge Foundation Germany, a non-profit organization working on transparency of public money. The Farmsubsidy.org project is currently unfunded and maintained by volunteers,^16^ with code released under open license with the intention to be maintained by the community.^17^ They publish the data exactly as published by national governments.^16^

We downloaded the raw CAP payment data for all available member states and years from FarmSubsidy.org on July 15, 2019, using the Linux command $wget -r https://data.farmsubsidy.org/latest/ (see instructions for download and for setting up the file structure in the readme file on our GitHub, https://github.com/kanicholas/CAP-farm-payments). Note that files are compressed and need to be extracted; we used Archive Manager on Ubuntu.

There were wide variations in the raw CAP payments data reported by member states (summarized in Table S1). Here we provide an overview of the assumptions we made to harmonize the data between countries (see supplemental information for full details).

We built our database using data from 2015; where data from 2015 were not available or were incomplete, we used the closest available year (2014 for Denmark and 2016 for Bulgaria, Sweden, and the Czech Republic). Although most member states list the payment year in their raw data, which was repeated in the file name, they do not clarify how years are reported. The CAP financial year runs from October 16 to October 15, with the payments published the following year. We assume that data files stating a year of 2015 refer to the majority year the payment was made (as stated by Germany on their transparency website, where searching 2018 is stated to apply to payments made from October 16, 2017, to October 15, 2018).^18^ Thus, we believe the majority of our data report spending undertaken from October 2014 to October 2015 and reported in the spring of 2016. We attempted to match all payments listed to the standard list of EU measures and converted all payments to euros. We classified about 1% of payments as from national rather than European funding (see supplemental information).

We used a combination of postal codes matched to NUTS3 regions and manual matching to obtain a standardized geolocation for each payment entry. In total, we successfully geolocated 83% of the payments in our database. We could not geolocate payments for nine countries where postal codes were not provided in the raw data, nor for about 19% of payments in Sweden that do not follow NUTS3 borders. In total we were unable to geolocate about €9 billion in total payments beyond the national level (about 15% of total payments in our dataset). In addition, we were unable to match 2% of reported locations to postal codes. This 17% of the dataset is thus geolocated to the national, NUTS0 level. To harmonize measure names across countries, we created a master Rosetta Stone file (Table S2) where we aligned names for the CAP payment measures given by member states in their national language to a common English language standard label. For the standard label, we used the “Description of Measures” published by DG AGRI, sometimes cited as Ares (2018),^19^ which lists 102 individual measures (27 in Pillar I and 75 in Pillar II), drawn from 10 different pieces of underlying regulations (listed in Table S3). Meta-data on the structure of the Rosetta Stone are given in Table S4.

Fourteen countries reported a standard measure identifier, such as the Roman numeral for the measure name in Ares (2018), that made matching measure names straightforward (see Table S1). For the remaining 13 countries, matching measure names required a combination of machine translation and native speaker assistance, research on national agency websites, and direct contact with national offices and country experts. Where judgment was required to match a reported measure name with the appropriate standard label, we developed a classification system for assessing the certainty of our match. We used the full dataset (all levels of match certainty) for analysis, but report the certainty level of matches by country and measure name for most of the measures in Table S5 in case others wish to have a more stringent cutoff. Four of the regulations underlying the 102 measures expired during the 2014–2020 CAP period, but remained valid for payments through 2015,^20^ so member states used a mix of old and new terminology in reporting the payments in our dataset. See Table S6 and notes in the Rosetta Stone regarding additional possible matches where short or ambiguous wording was reported for measure names.

### Data records

We have created two sets of data records using the methods described above, both of which are structured to reside in the “Output” folder on our GitHub (see Figure 1). First, we have created a “Translated country file” for each of the raw country data files. This maintains the original raw data for each country (every payment reported in the raw transparency data, e.g., over 3 million records for Poland, including any personal identifying information of recipients where such data were originally reported by member states) and appends to it additional columns to facilitate analysis, including standardizing to translated measure name, adding country and year (if missing), converting currency to euros, and adding the NUTS3 region (Figure 1).

These 27 translated country data records were then further processed to create the condensed year data record, which shows the payment amounts spent on each CAP measure for each NUTS3 region for the given year. For convenience, the resulting condensed files from running the workflow described in Figure 1 are provided in Tables S7–S9 for 2014, 2015, and 2016, respectively. These three files are themselves the input to the technical validation, as described below.

### Technical validation

The factors in public data reporting and curation that made this dataset difficult to generate also made it difficult to validate, namely, the impermanent availability of the raw data; the lack of coherent, centralized reporting that covers all CAP spending; the tendency to report total funding received during the 7-year CAP period rather than by year; the failure to consistently distinguish between EU and national funding in reporting payments; and the lack of accessible data showing payments by measure or by location finer than member state. Based on the current state of public data reporting on CAP spending, described below, we believe we have used the best available validation data, but we were not able to identify a publicly available source of information against which to comprehensively validate our data. Nonetheless, based on our validation efforts we are confident that these data represent the best currently publicly available data on CAP spending across the EU. We hope that the publication of our dataset spurs greater inquiry and transparency for the member states and the EU to report these data in a directly usable format (broken down by year, unique CAP measure ID, measure name, and location, including postal code), as we detail in the recommendations below.

### Original data validation

Because the transparency legislation requires data to be available for only 2 years, it was no longer possible to download the original data from the member states for 2015 for validation; we thus rely on the accuracy of the data scraped and stored by Farmsubsidy.org. Please see R code on the GitHub to read in the translated and condensed files (allocating payments to NUTS3 regions by CAP measure for all member states, using the years noted above) and perform the validation analysis and produce the figures and tables described in this section.

### Reporting of CAP spending across the EU

The EU reports annual spending in its expenditure and revenue data under Section 2, “Sustainable Growth: Natural Resources.”^21^ Spending is reported in the broad category of either Pillar I (European Agricultural Guarantee Fund [EAGF], line item 2.0.1) or Pillar II (European Agricultural Fund for Rural Development [EAFRD], line item 2.0.2), but a finer breakdown by the 102 measures under these broad categories is not available.

A broad comparison between our data and EU reported spending at the pillar level confirms very close agreement. Of the 90.5% of payments we were able to attribute to measures, our data total €38.9 billion for Pillar I and €16.0 billion for Pillar II for the years used (centered around 2015, with four countries using data from 2014 or 2016 as noted above). For the same countries and years, Eurostat reports €40.0 billion spending in Pillar I and the Commission reports €15.6 billion in spending for Pillar II. These totals are broadly in line with the budget and spending during the 2014–2020 CAP period. In our dataset, we were able to identify only a handful of payments made under measures from national as opposed to EU funding (totaling €0.62 billion, roughly 1% of our payment total) (Table S10), which could partly explain the difference between our total and that from the Commission.

Eurostat administers data on “subsidies on production” (item code 25000) in their “Economic accounts for agriculture by NUTS2 regions” (Table S11). However, after repeated requests for Eurostat support during 2018 and 2019, it remained unclear to us what these data actually represented in relation to CAP spending. The Eurostat “subsidies on production” total just over €47 billion for 2015 at the NUTS0 (member state) level, but many NUTS2 regions and even several member states contain no data in this table.

**Figure 2:**
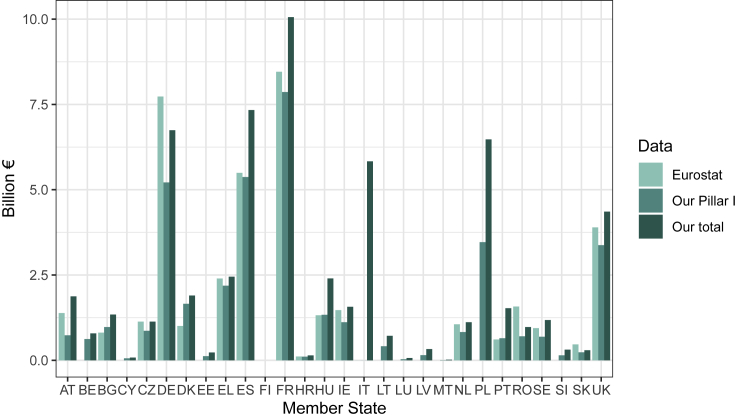
Comparison of farm payments for production reported by Eurostat with the payments in our database Eurostat payments reported are shown in light green, with our payments shown in both medium green (Pillar I) and dark green (total payments, Pillar I + Pillar II). Payments are for the years reported in Table S1 (2015 except for four countries.)

We compared the payments reported by Eurostat for each member state with our data, finding generally good agreement, although the payments in our dataset were generally slightly higher than those reported by Eurostat, with the exception of Denmark (Figure 2; Table S12). Our data generally show the expected pattern that Pillar I comprises the majority (approximately three-quarters or more) of the total CAP spending, with the notable exceptions of Austria, Hungary, and Poland, where about half of total spending in our data came from Pillar II.

### Reporting of CAP spending by member states

Distinguishing the purpose for each measure of reported funding is particularly difficult for Pillar II at the member state level, because reporting is spread out over many different venues, often aggregated across a 7-year CAP period rather than broken down annually, and often national funding sources are not clearly distinguished from EU funding (see supplemental information for more details).

The four member states who receive the most funding from Pillar II, EAFRD funding across the 7 years of the 2014–2020 CAP, are France (€11.4 billion), Italy (€10.4 billion), Germany (€9.4 billion), and Poland (€8.7 billion).^22^ Thus, these member states are especially relevant for analysis, and their reporting quality is of particular concern for clarification and improvement.

**Figure 3:**
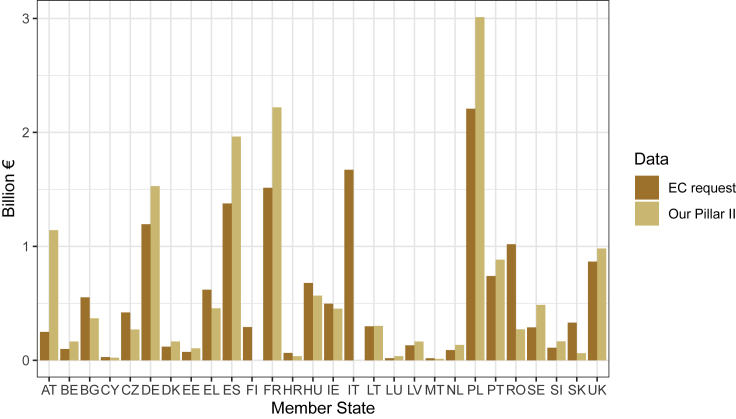
Comparison of Pillar II payments from 2015 reported by the European Commission and the Pillar II payments in our database Data from the Commission were obtained upon request from the ESIF Open Data Platform (see text and Table S10). Note that European Commission data are all from 2015, and our data are for the years reported in Table S1 (2015 except for four countries.)

Overall agreement for expenditure by measure and member state under Pillar II was generally reasonably close between our data and the data obtained upon request from the European Commission via the ESIF Open Data Platform (personal communication, May 14, 2020) (Figure 3). However, our data were notably higher than that from the Commission for Austria, Spain, France, and Poland (Figure 3). Here the Commission reported Pillar II spending in Italy of about €1.7 billion, which would fit reasonably well, as about 30% of our total of €5.8 billion (which we were unable to analyze by measure, as the raw data reported only the measure name “Total”). Note that data for this figure came only from 2015 from the Commission, but from 2014 or 2016 for four countries in our dataset as noted above.

From comparing our data with the most detailed data available from official sources, we find generally good agreement, with our data overall showing slightly lower payment values than reported by the European Commission broken down by measure in Pillar II, and slightly higher values than reported by Eurostat for all measures at the NUTS2 level. This is consistent with our inability to successfully match all payments to measures in the first case (9.5% of our payment data remained unmatched to measure) and to regions in the second (17% of payments could be geolocated only to the national level).

**Figure 4:**
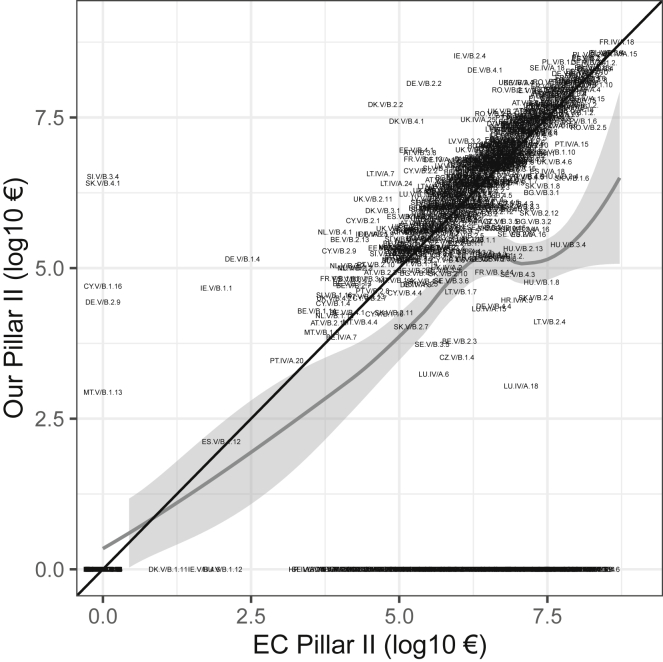
Comparison of Pillar II payments from 2015 reported by the European Commission and the Pillar II payments in our database, by measure and member state Data from the European Commission (EC) were obtained upon request from the ESIF Open Data Platform (see text and Table S10). Each of the 832 data points represents a unique PII measure in each country (see Table S13 for 55 negative values that were not plotted due to log transform scale). The black line is a one-to-one line; all points would lie on this line if the two datasets were identical. Points above this line are where our dataset reported higher values than the EC, and points below this line are where the EC data were higher. The gray line is a lowess curve, with 95% confidence interval shaded in light gray, indicating that overall, the EC data reported higher payment values than our data. The EC data also report spending under some measures in some countries that were missing or zero in our dataset (shown as values along y = 0). Note that EC data are all from 2015, and our data are for the years reported in Table S1 (2015 except for four countries.)

We analyzed agreement between our data for Pillar II spending and the data sent by the Commission for a set of 832 specific measures within countries, finding generally good agreement (most points lie close to the one-to-one line in Figure 4; Table S13). All points would be on the one-to-one line in Figure 4 if there were perfect agreement between our dataset and that of the European Commission. The gray lowess line indicates that on average, European Commission payments are reported as slightly higher than our data, especially as spending recorded by the Commission increases. This trend is caused largely by some of our data containing zeros or missing data where the Commission has recorded spending.

Taken together, the results of this analysis indicate that our independently collected and transparently constructed dataset is reasonably close to the data used by the European Commission, although not complete for all measures in all countries. However, there are some specific countries where Pillar II measures in our dataset are either substantially more (above the 1:1 line, such as SI.V/B.3.4) or substantially less (below the 1:1 line, e.g., LU.IV/A.18) than reported by the Commission. Although small in the overall CAP budget, if undertaking detailed analyses at the country or measure level, one should scrutinize such payments with caution.

**Figure 5:**
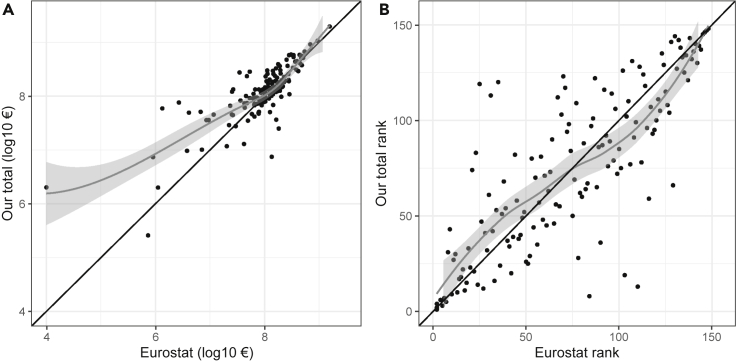
Comparison of CAP payments between Eurostat and our data at the NUTS2 level Comparison of CAP payments from 148 NUTS2 regions that were common between our data and those of Eurostat. The black line is a one-to-one line; all points would lie on this line if the two datasets were identical. Points above this line are where our dataset reported higher values than Eurostat, and points below this line are where the Eurostat data were higher. Note that three data points were excluded from (A), with zeros for Eurostat. Also note that Italy NUTS2 regions were all excluded with all zeros in Eurostat. The gray line is a lowess curve, with 95% confidence interval shaded in light gray. Its position above the one-to-one line indicates that for a few NUTS2 regions, our dataset was higher than that of Eurostat, but the two converged closely at higher payment values. (B) displays the rank order of the NUTS2 regions; the close agreement between the one-to-one and the lowess lines indicates a good agreement.

At the NUTS2 level, we were able to compare total CAP payments for 148 NUTS2 regions that were shared between our data and Eurostat. We found generally good agreement, with most points lying close to the one-to-one line, especially at higher payment values, where the gray lowess line converges with the one-to-one line (Figure 5A). This agreement was reinforced by the analysis of the rank order of these 148 NUTS2 regions between our data and Eurostat, showing close overlap (Figure 5B).

### Missing data and uncertainties

Overall, we were able to successfully match most payments from most countries both to the measure they supported (90.5% of payments) and to their spatially explicit geographic location within a country (83% of payments; most of the unmatched were in countries that did not report postal codes or other location information). Note that the vast majority of countries where measures were listed were able to be matched, as Italy listed no measures and comprised nearly 10% of the total payments in our database. See Python code “error_percentages.py” for the euro amount of payments unmatched, and “error_list.py” for the names of reported measures unmatched, on our GitHub. A country-level breakdown of the percentage of total funding within the year studied matched to both measure and location is shown in Table S1.

For many countries, less than 1% of payments were unmatched to either a measure or a location. More than 98% of all payments within a country were successfully matched to a measure, except in Greece (7.9% of payments unmatched to measure, due to high payments to six measure names not matched to the master list), Latvia (4% unmatched), and Denmark (4% unmatched to measure, due to errors in reported measure names); see Table S1. Other than the nine countries who did not report postal codes and were therefore 100% unmatched to a NUTS3 region, the only countries with over 2% of payments unmatched to location were Sweden (19.1% unmatched to NUTS3, due to non-overlap between postal codes and NUTS3 regions in Sweden); the Netherlands (13.4%), France (4.7%), Slovakia (4.4%), Italy (4.0%), and Malta (3.6%).

A number of countries had specific errors or issues with their measure formatting that required special processing or analysis. In brief, common errors included double entry of both total payments and subtotals for the same recipient (Latvia); ambiguous entries, such as article numbers that could apply to multiple measures (Denmark); reporting of old/expired measures under the previous CAP (e.g., Estonia); measure names not reported at all (Italy); and repeated entries for the same measure name, with variations in punctuation or spelling (e.g., Denmark, Romania), among others. See details about how country-specific issues were handled in the Python code and in Table S1, column “Errors and uncertainties in raw data.” This column could be used to inform member states of key areas to focus on for improvement in reporting.

Although we believe this dataset represents a substantial step forward in transparency of EU budget spending, data users should take care to understand the assumptions made. In particular, as described above and in the supplemental information, areas for attention include distinguishing national versus EU measures and the certainty of measure matches to the master list due to ambiguity in the raw data. Ambiguity in matching measures to the master list could arise both from how member states reported measures and from the existence of multiple measures dealing with the same topic. For example, a member state reporting the measure name “Advisory services” could apply to at least three measures; see the supplemental information. Further, there exist multiple measures dealing with the same topic (see Table S6).

For detailed analyses of specific measures, users should consult the “Notes” column by country in the Rosetta Stone (Table S2) and the match certainty rating in Table S5. Within Pillar II, given the overlapping intents and names between measures, it is probably most accurate to combine payments within similar measures for analysis by broad purpose (e.g., using Table S6), rather than focusing in detail on individual measures.

## Discussion

### Potential reuse value

We have undertaken to harmonize existing data reported by individual member states on public spending on the CAP, which have previously been very difficult to access in a way that facilitates the analysis and comparison essential for transparency. These data are important and relevant for researchers; policy makers; non-governmental organizations working with the Sustainable Development Goals, environmental stewardship, and other policy goals; and journalists reporting on public spending and government oversight, as well as the EU member states themselves and their citizens.

Given the high public interest in these data as the CAP reform discussions are ongoing, as well as extensive and ongoing calls for increased transparency of CAP spending, we believe the reuse potential for these data is high. This is especially the case when competing priorities highlight the need to use public resources wisely in pursuing urgent social goals such as sustainable food production, rapidly reduced climate pollution and enhanced natural carbon sinks, and biodiversity conservation. Despite the huge amounts of CAP spending, lack of suitable data at the appropriate time and scale is hindering effective evaluation of CAP measures in relation to their goals.^23^

We hope that this data harmonization effort can be carried forward to support more transparent and harmonized reporting by member states in the coming CAP spending period 2021–2027 to support ongoing analysis and collaboration toward achieving Europe's policy goals for sustainable agriculture.

We also see high potential to conduct further analyses with existing spatial data, now that these CAP payments have been made spatially explicit for the first time. For example, it would be interesting to analyze trends in social and environmental agricultural indicators related to the goals of the CAP, compared with payments made. (See Scown et al., 2020, for an analysis of these CAP payments compared with income, greenhouse gas emissions, and high-nature-value farmland location.)^11^ We note that the CAP dataset documented here can be analyzed in conjunction with a previously published dataset of 127 variables relevant for agriculture and the Sustainable Development Goals in Europe, such as greenhouse gas emissions from agriculture, water abstraction, and rural risk of poverty.^24^

### Recommendations

The difficulty we encountered in creating this harmonized dataset, and the remaining gaps and uncertainties in the data, demonstrates the need for common-sense reforms to streamline CAP payment reporting and data curation. Here we echo previous calls for EU member states to release data “according to a common format so that it is possible to analyse the data in a meaningful way across the European Union.”^16^

Below we offer recommendations to make the CAP payment data reported by member states more standardized to facilitate analysis and transparency. Some of our recommendations are very basic (consistently following existing regulations and good practices in data curation), while others would require updates or changes in current practices, but would offer substantial benefits to public transparency. These recommended improvements to data reporting would enable detailed analyses of CAP spending, which could inform and improve the overall performance of the CAP. These recommendations could be implemented at various levels, for example, in guidance for member states on reporting their strategic plans under the post-2021 CAP.

### Recommendations for future improvement in CAP data reporting

Most fundamentally, we recommend that future member state reporting of CAP spending require the following documentation to be reported through each country's data transparency portal:

• Member state payment reporting should include a column with the standardized EU-wide measure name and associated unique identifier for each measure, alongside the member state name used for the measure name to minimize errors. Further, member state reporting should include a column noting funding source for payments made (e.g., distinguishing payments made from the EAGF for Pillar I, from the EAFRD for Pillar II, and from national sources).
• A dictionary file that lists the measure names reported in the native language/format, referenced to a standard list of measure names and unique identifiers provided by DG Agri, should be included. The Rosetta Stone document provided in this paper (Table S2) could serve as an initial template to do so, although it would need to be updated for the measures adopted in the 2021–2027 CAP.
• A document listing the names of and briefly describing any additional national measures reported in the payment data (those not included in the standardized EU measures) should be included.
• The name of the agency and department responsible for curating the payment data, as well as a contact person, should be clearly specified, and their contact information for questions about the dataset should be provided.
• Meta-data should be provided for the whole dataset, including an explanation of negative values.

To facilitate analysis and minimize errors, the *format of data reported* should always include the following elements and consistently follow data curation conventions:

• In addition to any data preferred to report by the reporting member state, CAP reporting should always include the following data: country name, year, standardized EU measure name and identifier, recipient identifier (name *or* recipient ID, with unique European identifier for legal entities), recipient postal code, payment amount, currency used to report payment amount (if data were collected in a currency other than euros and converted to euros, the exchange rates used to convert to euros should be reported in the meta-data).
• A column to distinguish the source of payments made (EU or member state funds and their proportional contributions) should be included.
• Identifying data (measure name, ID, recipient name, etc.) should be completely filled in for each and every row of payments reported (not left blank under headings assumed to be carried down until a new entry appears, which hinders analysis). Where values are zero or not applicable, appropriate codes should be used and noted in the meta-data.
• Measure names and other text responses should be reported from a standardized list or pull-down menu, not entered by hand, to reduce the frequency of duplicate and erroneous measure names.
• When numbering is used as part of measure names, it should be done using unique consecutive numbers (such as 001, 002, …). Sometimes current numbering conventions yield non-sequential sort orders, such as measures in Denmark that mix numbers with names to label their measures; sorting them yields 1, 10, 11, 12, … 2, 20, 21, 22, ….
• Validation should be carried out by the reporting agency responsible before reporting data, to minimize errors. That is, the data should be totaled and reconciled with official statistics to ensure all payments have been accounted for, and standardized, unique measure names and ID numbers should be used to ensure every payment reported can be uniquely associated with its recipient and purpose, and to avoid errors in measure names.

Additional suggestions to improve data reporting (beyond current mandates) are as follows:

• Payment data would be much more usable in spatial format if postal codes were required to be reported (current regulation requires postal codes to be reported “where available”). Because they encode geospatial information, postal codes are much easier to link to open-source spatial databases that allow meaningful analysis than the currently used geospatial identifier, municipality.
• Member state reports would be much more useful if they directly reported NUTS3 region (and stated which NUTS3 version was used in the meta-data), since the documents available from Eurostat did not always convert postal codes to NUTS3 regions with high accuracy. This would enable detailed spatial analyses of CAP payments against other agricultural statistical information, for example, from Eurostat.
• Member states should make it possible not just to search by specific criteria, but also to directly download the full year's payment data from their websites (as a few countries have already done).
• We recommend implementing a unique European identifier for legal entities to help match recipients across countries and avoid duplicate entries. (The Open Knowledge Foundation Germany notes that the US and Mexican governments publish unique recipient ID codes that allow tracking the same recipient over different years and different datasets.)^16^ A unique identifier would also help address privacy concerns.

### New suggestions for reporting administration

Current legislation requires each member state to report its payment data on its own websites. If member states follow the recommendations above, their reported data will be much more usable. However, stronger guidance and coordination at the EU level would make the data much easier to analyze than downloading it from 27 separate websites. At a minimum, as noted above, the appropriate EU agency, such as DG AGRI, should produce a spreadsheet template with standardized measure names against which each member state should submit a dictionary file mapping how its reported measures map to the master measure names (essentially making the Rosetta Stone that we have created from scratch here the standardized reporting framework). Centralized curation of the data would likely improve accuracy and accessibility and make evaluation against result and impact indicators possible. It would also facilitate analysis if the EU made geospatial data on postal codes (.shp files) open source (they are currently proprietary). It would be a great help if centrally reported data were available in data (spreadsheet) format by year and pillar (instead of 118 separate PDF files for rural development programs across 2014–2020).

### Future research

For further analysis of past CAP spending, it would be helpful to expand the work done here to align measures reported during the entire 2014–2020 CAP (beyond 2015) with the standardized measure list. However, acquiring historical CAP spending data from member states is a challenge, since they are required to make the data available for only 2 years, after which most countries seem to remove older data from their transparency websites. Some additional historical data on country-level CAP payments are available from Farmsubsidy.org, although they are not able to archive all years given their current all-volunteer status. With additional resources, further research could be done to scrape and archive the data reported by member states for the current reporting period. Going forward to the CAP starting in 2021, we urge the European Commission and member states to follow the recommendations above so that such extensive compilation and harmonization will not be necessary to reveal how public money is being spent.

## Experimental procedures

### Resource availability

#### Lead contact

Further information and requests for resources and reagents should be directed to and will be fulfilled by the lead contact, Kimberly Nicholas (kimberly.nicholas@lucsus.lu.se).

#### Materials availability

Note that the full raw data files for CAP payments were originally published according to EU transparency law by member states and archived by the Open Knowledge Foundation Germany (https://data.farmsubsidy.org/latest/), and the code begins with extracting the raw files from that archive.

#### Data and code availability

Our Python and R scripts, instructions for accessing the raw data, and associated output data files are available on GitHub at https://github.com/kanicholas/CAP-farm-payments.

## Supplemental information

Document S1. Supplemental experimental procedures

Document S2. Tables S1–S13

Document S3. Article plus supplemental information

## Footnotes

Supplemental information can be found online at https://doi.org/10.1016/j.patter.2021.100236.
