# Supplementary material for: A harmonized and spatially explicit dataset from 16 million payments from the European Union's Common Agricultural Policy for 2015
Source: Patterns (N Y). 2021 Apr 9;2(4):100236. doi: 10.1016/j.patter.2021.100236 (PMC8085596; doi:10.1016/j.patter.2021.100236)
Supplement: Document S1. Supplemental experimental procedures [file mmc1.pdf]

Patterns, Volume *2*

Supplemental information

A harmonized and spatially explicit dataset from 16 million payments from the European Union's Common Agricultural Policy for 2015

Kimberly A. Nicholas, Frida Villemoes, Edmund Aristid Lehsten, Mark V. Brady, and Murray W. Scown

Supplemental Experimental Procedures

1. *Data Sources*

1.1. CAP Spending Reporting by EU Member States

The CAP spending data originally came from EU Member States, who are obligated to report spending to comply with the EU's principle of transparency, including financial regulations adopted in 2012 to publish information on recipients of EU funds, and a 2013 regulation with specific obligations for publishing CAP payment recipients.

Specifically, in Article 111 of Regulation (EU) No 1306/2013,[1] Member States are required to report the following information on a single website for at least two years following publication: payment beneficiaries (first and last names of individuals, or full legal name of associations or companies); the municipality where the beneficiary is registered (and postal code "where available"); the amounts of payment corresponding to each measure; and "the nature and description of the measures" for both EU and Member State contributions.

Regarding data accessibility, the European Commission states that *"anyone who receives EU funding under the CAP is included on a publicly available list. This list is designed to promote transparency and trust in EU funding measures. However, the rules still need to strike a balance between the rights of the public to know how their money has been spent and the rights of individuals to protect their personal data. The data is therefore limited in its scope and only available for a set period. [...] It is available from the 31 May of the year after the payments were made. This information will be publicly available for two years after its publication."*[2]

Some CAP beneficiaries are exempted from reporting requirements when the benefits they receive are small, or may be anonymized in some cases. For example, Article 112 of Regulation (EU) No 1306/2013[3] exempts reporting requirements for beneficiaries who receive less than €1250 per year. Additionally, some countries give codes to recipients (although some codes consist of recipient names and locations) or anonymize their data in certain cases. For example, Denmark does not publish the municipality and postal code of anonymous beneficiaries if there are fewer than 10 beneficiaries within a municipality.[4] A few Member States list open data reuse policies on their websites. For example, the UK states that the CAP data are under an Open Government License for public sector information, where users are "encouraged to use and re-use, free to copy, publish, distribute and transmit the information."[5]

The European Commission maintains a webpage[6] with links to each country's CAP payments reporting website, where they state, "*To ensure full transparency, EU countries publish information relating to the beneficiaries of all common agricultural policy (CAP) payments on their national websites."* Each Member State currently maintains its own database for reporting CAP spending, all of which use different formats, with no universal standard for the "nature and description of measures." A few Member States have made it possible in recent years to directly download the full data from current years (e.g., Spain, UK, Hungary, and Portugal), but most national transparency websites only allow searching by specific recipients, measure name, or locations, or using pulldown menus, with results reported in html or displayed as only a few entries at a time (e.g., UK and Sweden). Thus, it is very difficult to get a comprehensive overview of where CAP spending went and for what it was intended.

1.2. Open Knowledge Foundation Germany & Farmsubsidy.org

Because each Member State has their own webpage for reporting CAP spending data, and each uses a different format, obtaining the raw data in a format suitable for analysis presents a significant challenge. We used raw CAP payment data curated by FarmSubsidy.org, which is a project of the Open Knowledge Foundation Germany, a non-profit organization working on transparency of public money. The Farmsubsidy.org project was started in December 2005 by three journalists. Over the years, project

members have worked to “file freedom of information requests on a national level and to clean, compile and present the obtained data on the new Farmsubsidy.org website.”[7] The stated aim of the Farmsubsidy.org project is to “…obtain detailed data relating to payments and recipients of farm subsidies in every EU member state and make this data available in a way that is useful to European citizens.”[8]

The Open Knowledge Foundation works to extract the data currently reported separately by Member States and make the raw data files available in one place. To do so, they have built data scrapers for each country website, which are released under open license with the intention to be maintained by the community.[9] The code for the farmsubsidy.org scrapers are available on GitHub; most were last updated between 2016-2018, although Cyprus was added in late 2019.[10]

Farmsubsidy.org states that they publish the data exactly as published by national governments: “Ultimately, the data available on this site is only as good as the data we have received from the governments. We do not change the data we have received from governments so if you think you have identified an error in the data you should notify the relevant government agency and, if possible, let us know too.”[11]

The Farmsubsidy.org project discloses their funding on their website, which states that most project funding was received between 2006-2010, with the last funding listed as €5,000 in 2014. Their website states “Currently this project is not funded, but maintained by volunteers.”[12]

1.3. Downloading data and data content

We built on the work already undertaken by farmsubsidy.org to use the data they had scraped and made available. We downloaded the raw CAP payment data for all available Member States and years from FarmSubsidy.org on July 15, 2019, using the Linux command
$wget -r https://data.farmsubsidy.org/latest/. These data can be searched on the farmsubsidy.org website by address given or by amount, but not by standardized measure (scheme) name or geolocation, because these features are not reported in the original data.

Each raw data file from farmsubsidy.org listed information potentially including recipient name and address, amount paid, currency, year, and CAP measure under which the payment was made. Not all Member States report all data. Header names were evaluated manually to be sure to include all relevant data (for example, Romania lists measure names (what other countries call “scheme” in their raw data) under the column heading “scheme_2”). See Table S1 for an overview of the content of the raw data files of CAP payments.

2. Data Availability by Country and Year

2.1. Countries and years included for CAP payments

Data on farmsubsidy.org were available for a range of years that varied by country, though most countries had data available for 2014-2017. We built our database using data from 2015; where this was not available, we used the closest available year (2014 for Denmark, and 2016 for Bulgaria and Sweden). Additionally, we present data from 2016 for the Czech Republic, because the 2015 data contained only about 5% of the data volume and 20% of the payment totals than the previous and following year, and was confirmed to be more than five times smaller than the Eurostat payment reported for 2015. Finland had data only for 2004-2013, during the previous CAP period, and was therefore not included. The data for Italy reports only “Total” for each payment rather than listing specific measures, so it was not possible to classify CAP payments in Italy by measure.

During data validation, we identified anomalously low payments for the Czech Republic in 2015 (leading us to realize the 2015 raw data was only about 5% as long as the files before and after, and we decided to use 2016 which had a full dataset). We note our data for Romania are about a third lower than the

payments from Eurostat, but Eurostat data also report 2015 as a year of substantially lower payments to Romania compared with 2014 or 2016, so we elected to keep our 2015 data.

2.2. National vs. EU measures

Although Article 111 of Regulation (EU) No 1306/2013[13] requires Member States to report payment amounts and "the nature and description of the measures" for both EU and Member State contributions, we did not find a consistent system for distinguishing EU and Member State contributions reported in the payment data. For the purposes of this paper we follow the EU terminology used in the legislation and refer to the various payments that Member States are permitted to make under the CAP as *measures* (which elsewhere are referred to more generally as policy instruments or payment schemes depending on the literature).

As a brief explanation for distinguishing European and Member State financing under the CAP, the CAP is divided into two programmes known as "Pillars": Pillar I, entirely financed by the EU[14] through the European Agricultural Guarantee Fund (EAGF), directs the majority of the CAP budget to support direct payments to farmers (71.3% of CAP spending for 2014-2020) and market measures (4.3%).[15] Pillar II finances the remaining 24.4% of CAP spending through the European Agricultural Fund for Rural Development (EAFRD).[16] Pillar II programs support rural development and environmental measures. They are co-financed by EU funds and regional or national funds.[17]

The European Commission states that funding reported under Pillar II includes both EU and national sources: "For funding from the European Agricultural Fund for Rural Development (EAFRD), the amounts published include both the money received from EU funds and from the EU country. This means that the reported amount reflects the total amount of public expenditure on the project."[18]

A few countries listed the funding source in the name of the measures (e.g., Hungary listed National, EAGF, or EAFRD), but most did not. Some reported only EU-funded payments; e.g., Denmark stated "National aid schemes without EU funds are not published".[19]

The farmsubsidy.org data scraped the raw CAP payment data exactly as reported by Member States,[20] which "may also contain non-EU national subsidies which are sometimes in the same database" (Stefan Wehrmeyer, personal communication).

In the absence of any information about national vs. EU funded support in the raw data, we assumed all measures listed were from EU funding, and matched all that we could to the standardized list of EU measures. We classified any measures listed to have national support (either in the measure name, or from information from the reporting agency) with the ID code National, rather than matching to a specific CAP measure (ca. 1% of total payments).

3. Payment values

3.1. Negative payment values

We included all payment values exactly as reported, including negative payment values, which comprised a small proportion of payments in a few countries. The German CAP payment agency Bundesanstalt für Landwirtschaft und Ernährung (Federal Agency for Agriculture and Food) states that positive amounts in the transparency portal refer to payments from the EU budget to beneficiaries, whereas when goods from a public intervention are sold, the price paid by the buyer for the goods appears as a negative amount, representing revenue for the EU budget.[21]

For nearly all countries, negative payments were non-existent or very small (less than 1% of total payments for the year). For instance, for the largest payment recipient, France, negative payments were about 0.4% of total payments in 2015). The only countries to have such payments be more than 2% were

Cyprus (2.7%) and Greece (13%, dominated by one very large negative payment of nearly €200 million). (See file "missing_money_percentages" on our GitHub.) We checked the current data on the Greek transparency website[22] and found 12,024 negative values reported from 2017, so we assume these payments were valid.

3.2. Payments between countries

In the first step of our Python code, payments are summed by country that reported receiving the payment (as the EU reports). But two countries (Belgium and the Netherlands) report payments to other countries within their national transparency reporting, perhaps indicating that the landowner lives abroad. In the second condensation step of our data processing (Figure 1), our Python code attributes all payments to the beneficiary country listed.

For example, payments listed in the raw data reported by Belgium as having a recipient in France would be added to our translated file for France, rather than Belgium. However, these international payments were only reported by Belgium and the Netherlands, and in both cases they were a small percent of the total country payment (about €2.6 million for Belgium, less than half of 1% of their total payments received; and about €13,000 for the Netherlands in 2015, compared with their total payments received of over €1 billion). See the code for "money_movement.py" on our GitHub.

4. Standardizing location and currency

4.1. Location

To obtain a standardized geolocation for each payment entry, we extracted postal codes given in the raw CAP payment data, and matched these to NUTS3 regions, developed and maintained by the EU and generally corresponding to the finest-scale level above municipalities, such as counties or provinces depending on country nomenclature. For example, NUTS3 units in France correspond to 101 Departments.[23] We used the 2013 version of NUTS3, which was appropriate for our 2015 data.[24] We downloaded conversion files from postal codes to NUTS3 regions using the postal code to 2013 NUTS3 regions correspondence table available from Eurostat individually for each country[25] (also found on our GitHub) and used the Python code to extract country and postal codes and match them to NUTS3 regions.

For data where postal codes in the raw data did not match to the available NUTS3 files, we manually matched them where possible by determining the classification system for each country and looking at maps of postal codes and NUTS3 regions to determine the coding pattern between postal codes, which are generally a subset of NUTS3 regions (see Python code, and descriptions in the column "How to match postal codes to NUTS" in Table S1). For most countries, this reduced the amount of payments that could not be geolocated to less than 2% of the total payments. However, Sweden's postal code numbering system[26] does not follow NUTS3 borders,[27] so 19.1% of payments in Sweden could not be linked with a NUTS3 region.

For ten countries (Bulgaria, Czech Republic, Estonia, Greece, Ireland, Lithuania, Luxemburg, Latvia, Romania, and Slovenia), postal codes were not given in the raw data. These countries did include a "recipient location" (usually city) but it was deemed too time-consuming to look up postal codes or otherwise couple them to NUTS3 regions (this could not be automated since shapefiles of postal codes are unfortunately proprietary). Luxembourg, however, consists of only one NUTS3 region, so all payments could be allocated to that NUTS3, leaving nine countries where we could not allocate payments to NUTS3 regions (see Table S1). These nine countries with payments only allocated to the national (NUTS0) level rather than finer NUTS3 spatial level represent about €9 billion in total payments (about 15% of total payments in our 2015 dataset).

4.2. Currency

For countries that did not state a currency of reported payments, we assumed payments were made in Euros (cross-checked with payment totals reported by the EU). Seven countries reported payments in a currency other than the euro. These payments were converted to euros using the average conversion rate for the year of payment. We used currency exchange rates reported by the European Central Bank Euro Reference Exchange[28] to obtain the average value of the given currency for the given year. Values were extracted in (currency) to euro, for January 1 to December 31 for the year of interest. All values are given in euros for the year reported (i.e., they are not converted further to a standardized year). Currency conversion rates are listed in the Python code.

Poland did not list a currency for its payments in the 2015 raw data, but its transparency website stated that payments are shown in PLN, and the total reported payments in the raw data for 2015 totaled 27 billion, whereas the true value was known to be around €6 billion, so we converted the reported payments from Poland to euro assuming they were reported in złoty.

5. Standardizing Payment Measures: Creating the "Rosetta Stone"

We digitized the PDF version of "Description of Measures" into a spreadsheet to consistently align measure names with associated legislation and names in national languages. This spreadsheet formed the basis of the "Rosetta Stone" we used to match measure descriptions across countries.

We added additional classification information compiled during our research to aid with both matching of measure names (our creation of the Rosetta Stone document), as original country scheme names sometimes contained reference to e.g., rural development program measure numbers, and to understand the purpose of each measure for further analysis. (See Table S4 for an overview of the structure of the Rosetta Stone document and the source of our classifications.) After this meta-data, the Rosetta Stone document consists of columns for each country with original scheme name reported, translated scheme name (where relevant), and notes on the matching criteria.

Following DG AGRI, we used the measure identifier (a combination of a Roman numeral and Arabic numeral ID) in the first column of the tables in "Description of Measures" to uniquely identify each of the 102 possible measures for the analysis, which is reproduced in the first column of Table S2. This ID cannot be directly related to legislation, but consists of two or three parts: the short heading for each section in "Description of Measures" (I, II, III, IV/A, V/B, VI/A, VI/B, VI/C and VI/D), and a row number (i.e., 1, 2, 3,…) or sub-table number and row number (i.e., 1.1, 1.2, …), such that III.2 is the ID for the second measure listed in section III and V/B.1.10 is the tenth measure in sub-table one of table V/B. Each measure is linked to the underlying legislation via the columns immediately following the ID: Regulation, Title, Chapter, Section, Article where relevant.

Matching the reported measure name to the master list was straightforward for the 14 countries that used the measure ID to report their measures (as noted in "Includes standard numbers of Measure Descriptions" column in Table S1). However, the remaining 13 countries did not use a standardized identifier for measure names (instead using a wide variety of short descriptions in local languages and/or numerical codes with a variety of meanings). This lack of standardization means it is impossible to use the raw data reported by Member States for cross-country analysis, which is why we undertook the harmonization to make the Rosetta Stone. To take a simple example, different countries reported the following names for a measure, all of which we matched to the first measure (I.1, Single Payment Scheme):

"Single payment scheme - title III"
"I.1 - Aide unique découplée à la surface (DPU)"
"I.1 Καθεστώς ενιαίας ενίσχυσης – τίτλος III (ΕΓΤΕ)"
"I.1"

“Guarantee Fund direct support: I.1, Single payment scheme - title III”

More details about the format and examples for each country reporting their measures are given in Table S1.

For those countries whose measures were not reported according to the measure identifier, we identified unique measure names from national languages and manually matched reported country measure names to the 102 individual measures of DG AGRI using a combination of machine translation, native language speakers, and input from national agriculture experts, as we describe in detail in the next sections.

5.1. Extracting unique measure names

We extracted all unique measure names for each raw country file for analysis in our database (N= 27, all of the EU-28 from 2015, except Finland who reported no data for 2015). We then manually matched measure names from countries to the master list of measures in the Rosetta Stone, using the measure numbers and/or descriptions given as follows.

5.2. Translation and uncertainty assessment

To match measure names used by each country, we created three columns in the Rosetta Stone document for each country: original country scheme name or code (pasted exactly from the raw data files), translated country scheme name (for non-English measure names, this contained the best translation of the original scheme name into English where relevant, preferring native speaker translations over automatic ones), and Notes justifying the basis of the match.

For measure names listed in a language other than English, we first used Google Translate to translate an Excel file containing all measure names from the original language to English, and then manually matched as many measures as possible using the translated names and combination of searching and manual matching with the master measure names.

We distinguished the following classifications for the level of certainty of the match between the national measure name with the master measure list, in order of greatest to least certainty:

Match: all information present, both words and numbers where present, was a strong match to the master list. In other words, both words and numbers matched the master list (if the measure consisted of both words and numbers), words matched the master list (if the measure name consisted only of words) and numbers match (if the measure name consisted only of numbers).

Words but not numbers match: when words from a national measure matched with the master list, but either did not contain numbers present in the master list, or contained numbers that differed from the master list. Where possible to identify, the meaning of the numbers was noted (e.g., when they referred to underlying legislation, article numbers, national measures, or other references).

Unique but imperfect match: the best available match, although some information between the original and master list was inconsistent.

Best of multiple possible matches: more than one match was possible, but enough information was given to support matching with a particular measure.

No match: it was not possible to align the original measure name with a master measure name, because the description given did not fit any of the 102 master measure descriptions from DG AGRI. All non-matches were double-checked against the raw data to ensure they had been imported properly, and that errors (e.g., measure names such as “-” or measure names that cut off in mid-sentence) had indeed been present in the original data and not introduced during our import or analysis.

Note that some measures were placed in both the category “Words but not numbers match” and “Best of multiple possible matches,” as they were matched with the best of a number of options, but the numbers did not match.

Note that most countries either had more measure names in their transparency reporting data than the 102 present in the master list (e.g., Hungary listed 274 unique measure names), or listed more than one national measure that was ultimately matched to the same “Description of Measures” name. In such cases, an overflow row was created in the Rosetta Stone, proceeding in alphabetical order by country, with one row per additional measure name. Thus, the first 102 elements of the Rosetta Stone document (rows 2-103, following the header) contain the first instance of a match for an original language scheme name across all countries, with potentially many matches across many countries for each row. Subsequent matches within a country start at row 103 in the Rosetta Stone and continue to row 795, thus there were 692 individual measures added after the main data, where each row lists only one country scheme name, and abbreviated information in the first columns to show the match with the master scheme name.

5.3. Native speaker assistance

For many countries, a substantial fraction of measures remained unmatched at this stage, and we sought the help of native language speakers to improve upon the translations suggested by Google Translate. In many cases native speaker assistance made it possible to make a successful match to align reported measures with the master list.

Native speakers were recruited by the first author on a volunteer basis in an email explaining the purpose of the study, requesting their help in translating into English the measure names that remained unmatched (typically 10-30 short phrases, requiring less than an hour of work), and promising acknowledgement in and a copy of the resulting scientific publication in exchange for their assistance. One native speaker (of Hungarian) was paid as a research assistant for approximately 10 hours of her time in researching Hungarian law as well as providing translations, which helped place many additional measures. We gratefully acknowledge the contributions of all of the native speakers in helping to create this dataset; please see the full list in the Acknowledgements.

5.4. Searching national agency websites

For all countries with measures still not matched to the master list at this stage, the first author searched national agency web pages for any additional information that might assist with matching measure names. In a few cases, it was possible to triangulate information that now appears on national transparency websites to assist in making further matches (e.g., when original language and English translations of measure names were both available), or to download a full list of measure names in their original language with enough information to make a match. These were noted in the relevant country “Notes” column of the Rosetta Stone file.

5.5. Contact with national offices and country experts

After following the procedure above, where any non-matches for measure names remained, the first author sought contact with national agricultural offices in charge of administering CAP payments. Contact information was found from following the links from the official Member States CAP transparency pages, as well as independent research, including seeking contact via the official payment agency Twitter account where available. These details were not always easy to find; to facilitate future contact for others, public email, webpage, and Twitter accounts are listed in Table S1.

The majority of contact attempts with national agricultural agencies to clarify measure names received no reply (contact by email and/or Twitter to the official contact listed for France, Romania, Hungary, Sweden, Lithuania, Bulgaria, and Croatia). Phone contact was successfully made with the UK and Germany, but attempts to follow up with the relevant expert were not successful to receive further information.

(However, contact was successfully made with a CAP expert at an NGO in Germany, who provided additional translations and confirmed matches of measure names to the standard EU list in the Rosetta Stone) (Christian Rehmer, personal communication, 2 March 2020). Twitter contact was successfully made with the agricultural agency in Ireland,[29] but the promised answer from the appropriate section was never received. In several cases, no contact information was readily available (for example, the Austrian CAP payments database website[30] was made by a marketing company; the government agency administering the payment was not clearly listed).

Replies to requests for measure information were received from five countries: Estonia, Latvia, Czech Republic, the Netherlands, and Germany. Representatives from the agricultural agencies in Czech Republic and the Netherlands sent a full list of native measure names aligned with the master list upon request, which enabled complete measure name matching. A representative from Latvia sent a list of measures aligned which enabled matching of all but eight measures (five of which translated as "Action" plus a number). Representatives from the agricultural agency and ministry in Germany provided helpful responses confirming our matches and clarifying the validity of funding periods for different measures. A representative from Estonia sent a match with the 2007-2013 CAP, which was not possible to translate to the current 2014-2020 CAP. Requests to do so did not receive a further reply.

5.6. Expiration and ambiguity of measure names

Complicating the analysis, four of the Regulations underlying the 102 measures expired during the 2014-2020 CAP period, but remained valid for payments through 2015,[31] so Member States in 2015 used a mix of old and new measure terminology in reporting payments. See the correspondence given by EU Regulation between measures in V/B. and IV/A (Table S6).

In Pillar I, Regulation EC 73/2005 (underlying the seven measures related to direct payments in Pillar I for 2007-2013, starting with Roman numeral I) expired in 2013 and was replaced by Regulation 1307/2013 (now with ten measures in Pillar I, starting with Roman numeral II). This means that for example, a country reporting a measure by the name "single area payment scheme" might refer to either I.2 or II.2, both of which use that terminology.

For Pillar II, Regulation EC 1698/2005 (underlying the 46 rural development measures in Pillar II starting with V/B) expired in 2013 and was replaced by the 25 Pillar II measures starting with IV/A associated with Regulation EU 1305/2013. The single measure on information and promotion VI/B.1 (Regulation EC 3/2008) was repealed and replaced by the same measure under Regulation EU 1144/2014 with ID VI/A.1.

Sometimes countries reported a measure using only a short, ambiguous label. For example, three measures relate to "advisory services": IV/A.2, "Advisory services, farm management and farm relief services"; V/B.1.4, "Use of advisory services by farmers and forest holders"; and V/B.1.5, "Setting up of farm management, farm relief and farm advisory services." When countries listed a measure using only a short descriptor such as "Advisory services," it is impossible to say with certainty which measure was indicated.

Finally, one of the two measures making up measure VI/C.1, the POESI measure, expired in 2013, but the measure remained active under the same ID (VI/C.1) with the subsequent regulation.

5.7. Grouping of measures

At the broadest level, we distinguished between measures in Pillar I (the first 27 listed in Table S2) and Pillar II (the last 75 measures), by a column indicating the corresponding funding source for the two Pillars (European Agricultural Guarantee Fund (EAGF) for Pillar I and European Agricultural Fund for Rural Development (EAFRD) for Pillar II) as the local-language version of this acronym was often used in measure names reported by Member States. For further analysis of measure purpose, please see measures identified as income support to farmers and as "environmental payments," which encompasses

all CAP measures which state in the measure wording the intention to principally benefit nature, the environment, climate, or to promote sustainable farming, see Table S2 in Scown et al. (2020).[32]

5.8. Finalizing matches

To finalize the Rosetta Stone was an iterative process involving updating the placement of measure name matches, running the Python code, examining remaining errors and non-matches reported, and repeating until the only remaining non-matches were genuine. Measure names identified as valid non-matches from the translation exercise (where no appropriate match could be found to the 102 measures) were left out of the Rosetta Stone. All other measure names were matched to the most appropriate measure. The Rosetta Stone contains the most current and up-to-date data justifying translations and matches in the Notes column for each country. Some measure names returned as non-matches appeared identical to existing measures already placed, but were added as additional rows in the Rosetta Stone, with measure names pasted exactly as they appeared, in order to obtain a match. Note that all measure names were converted to strings (since there were some measure names that consisted only of numbers) and that spaces and punctuation marks were stripped from the beginning and end of the string to facilitate matching.

6. Existing reporting of data used for validation

Each Member State submits one or more Rural Development Programs (there were a total of 118 Rural Development Programs for the 2014-2020 CAP[33]), which are available as individual PDF files[34] and not in a database format that would more easily support analysis of spending. Some of the countries that receive the most CAP funding have a large number of different rural development programs for different regions (30 in France, 23 in Italy, 19 in Spain, and 15 in Germany),[35] making it very difficult to examine rural development spending by Member State.

The European Network for Rural Development provides spending breakdowns for a subset of measures within Pillar II, but these are difficult to use for validation of one year of data, as they are reported as aggregated over four to seven years in individual PDF reports for Member States where available, for example for individual measures from 2007-2011[36] or for percentage of spending by measure for each Member State for 2014-2020.[37]

Member States tend to report their spending over the full CAP program period, making it difficult to get an independent report of spending for a specific year. For example, the country factsheets list total spending by Member State, including total finances available for the full funding period, but do not distinguish between measures. For example, Sweden's Rural Development Program 2014-2020[38] states that Sweden will use €4.3 billion of public money from 2014-2020 for rural development, of which they state €1.8 billion is from the EU budget and €2.5 billion is national co-funding,[39] but a further breakdown of Pillar II spending is not given.

Financing for rural development consists of co-financed money from the European Agricultural Fund for Rural Development (EAFRD, €99.6 billion for the 2014-2020 multiannual financial framework, averaging €14.2 billion per year over the seven-year spending period) and regional or national public funds varying by region and measure (consisting of €50.9 billion in regional or national co-funding, and a further €10.7 billion of purely national funding, totaling €61.6 billion),[40] or an average of €8.8 billion per year. Thus, on average across the seven years, €23 billion in total is spent annually on rural development, with about two-thirds coming from the EAFRD and one-third from regional or national co-funding.

References

[1] European Parliament and Council (2013). REGULATION (EU) No 1306/2013 OF THE EUROPEAN PARLIAMENT AND OF THE COUNCIL of 17 December 2013 on the financing, management and

monitoring of the common agricultural policy and repealing Council Regulations (EEC) No 352/78, (EC) No 165/94, (EC) No 2799/98, (EC) No 814/2000, (EC) No 1290/2005 and (EC) No 485/2008. https://eur-lex.europa.eu/legal-content/EN/TXT/HTML/?uri=CELEX:32013R1306&from=en

[2] European Commission (Undated). Common Agricultural Policy: Controls and Transparency. https://ec.europa.eu/info/food-farming-fisheries/key-policies/common-agricultural-policy/financing-cap/financial-assurance_en

[3] European Parliament and Council (2013). Regulation (EU) No 1306/2013 of the European Parliament and of the Council of 17 December 2013 on the financing, management and monitoring of the common agricultural policy and repealing Council Regulations (EEC) No 352/78, (EC) No 165/94, (EC) No 2799/98, (EC) No 814/2000, (EC) No 1290/2005 and (EC) No 485/2008. https://eur-lex.europa.eu/legal-content/EN/TXT/HTML/?uri=CELEX:32013R1306&from=en

[4] Miljø-og-Fødevareministeriet Landbrugsstøtte (Undated). See text under "Hvad bliver ikke offentliggjort" ("What will not be published?") https://lbst.dk/tilskud-selvbetjening/soeg-i-registre/modtagere-af-eu-stoette/landbrugsstoette/#c33575

[5] The National Archives (Undated). Open Government License for public sector information. http://www.nationalarchives.gov.uk/doc/open-government-licence/version/3/

[6] European Commission (Undated). Beneficiaries of CAP funds: Beneficiaries by country. https://ec.europa.eu/info/food-farming-fisheries/key-policies/common-agricultural-policy/financing-cap/controls-and-transparency/beneficiaries_en

[7] Open Knowledge Foundation Germany (Undated). Farmsubsidy.org at a glance. https://farmsubsidy.org/about/

[8] Open Knowledge Foundation Germany (Undated). Farmsubsidy.org FAQs. https://farmsubsidy.org/faq/

[9] OpenSpending/Drewes, H. (2014). Farmsubsidy.org Developer Documentation. https://farmsubsidy.readthedocs.io/en/latest/

[10] Wehrmeyer, S. (Undated). Scrapers for FarmSubsidy data in the member states. https://github.com/openspending/farmsubsidy-scrapers

[11] Open Knowledge Foundation Germany (Undated). Farmsubsidy.org FAQs. https://farmsubsidy.org/faq/

[12] Open Knowledge Foundation Germany (Undated). Farmsubsidy.org FAQs. https://farmsubsidy.org/faq/

[13] European Parliament and Council (2013). Regulation (EU) No 1306/2013 of the European Parliament and of the Council of 17 December 2013 on the financing, management and monitoring of the common agricultural policy and repealing Council Regulations (EEC) No 352/78, (EC) No 165/94, (EC) No 2799/98, (EC) No 814/2000, (EC) No 1290/2005 and (EC) No 485/2008. https://eur-lex.europa.eu/legal-content/EN/TXT/HTML/?uri=CELEX:32013R1306&from=en

[14] European Parliament (2020). Fact sheets on the European Union - Second pillar of the CAP: Rural Development Policy. https://www.europarl.europa.eu/factsheets/en/sheet/110/second-pillar-of-the-cap-rural-development-policy

[15] European Parliament (2020). Financing of the CAP. https://www.europarl.europa.eu/factsheets/en/sheet/106/financing-of-the-cap.

[16] Pe'er, G., et al. (2017). Is the CAP it for Purpose? An Evidence-Based Fitness-Check Assessment. German Centre for Integrative Biodiversity Research (iDiv). https://eeb.org/publications/53/farming/17992/is-the-cap-fit-for-purpose-a-rapid-assessment-of-the-avidence-dr-guy-peer-and-sr-sebastian-lakner-preliminary-summary-of-key-outcomes-june-2017.pdf

[17] European Parliament (2020). Fact sheets on the European Union - Second pillar of the CAP: Rural Development Policy. https://www.europarl.europa.eu/factsheets/en/sheet/110/second-pillar-of-the-cap-rural-development-policy.

[18] European Commission (Undated). Ensuring the correct payment of CAP funds. https://ec.europa.eu/info/food-farming-fisheries/key-policies/common-agricultural-policy/financing-cap/controls-and-transparency

[19] Miljø-og-Fødevareministeriet Landbrugsstøtte (Undated). See text under "Hvad bliver ikke offentliggjort" ("What will not be published?") https://lbst.dk/tilskud-selvbetjening/soeg-i-registre/modtagere-af-eu-stoette/landbrugsstoette/#c33575

[20] Open Knowledge Foundation Germany (Undated). Farmsubsidy.org FAQs. https://farmsubsidy.org/faq/

[21] Bundesanstalt für Landwirtschaft und Ernährung (Undated). Empfänger EU-Agrarfonds – Suche. https://www.agrar-fischerei-zahlungen.de/agrar_suche_hilfe.html

[22] Hellenic Republic Ministry of Reconstruction of Production, Environment, and Energy (2015). CAP Beneficiaries Payments. https://transpay.opekepe.gr/

[23] Wikipedia (Undated). Nomenclature of Territorial Units for Statistics. https://en.wikipedia.org/wiki/Nomenclature_of_Territorial_Units_for_Statistics

[24] Eurostat (Undated). History of NUTS. https://ec.europa.eu/eurostat/web/nuts/history.

[25] Eurostat (Undated). Postcodes and NUTS, https://ec.europa.eu/eurostat/web/nuts/correspondence-tables/postcodes-and-nuts.

[26]GfK GeoMarketing (Undated). 2-digit postcodes Sverige. https://upload.wikimedia.org/wikipedia/commons/e/ee/2_digit_postcode_sweden.png

[27] Eurostat (2018). SVERIGE - NUTS level 3. https://ec.europa.eu/eurostat/documents/345175/7451602/2016-NUTS-3-map-SE.pdf

[28] European Central Bank (Undated). Euro foreign exchange reference rates. https://www.ecb.europa.eu/stats/policy_and_exchange_rates/euro_reference_exchange_rates/html/index.en.html

[29] Nicholas, K.A. (February 24, 2020). https://twitter.com/KA_Nicholas/status/1231969619465900041?s

[30] Transparenzdatenbank EU (Undated). "Information zur Veröffentlichung von Zahlungen im Rahmen der Gemeinsamen Agrarpolitik der EU" https://www.transparenzdatenbank.at/

[31] EAFRD (Pillar II) payments for the programming period 2007-2013 were allowed until December 31, 2015, according to Art. 71 para. 1 VO (EU) Nr. 1698/2005.

[32] Scown, M.W., Brady, M.V., and Nicholas, K.A. (2020). Billions in misspent EU agricultural subsidies could support the Sustainable Development Goals. *One Earth 3*(2): 237-50. 10.1016/j.oneear.2020.07.011.

[33] European Commission (Undated). Rural development programmes 2014-2020. https://ec.europa.eu/info/sites/info/files/food-farming-fisheries/key_policies/documents/rdp-2014-20-list_en.pdf

[34] European Commission, Directorate-General for Communication (Undated). Rural development programmes by country. https://ec.europa.eu/agriculture/rural-development-2014-2020/country-files/

[35] European Commission (Undated). Number of Rural Development Programs per country (total of 118). https://ec.europa.eu/info/sites/info/files/food-farming-fisheries/key_policies/documents/number-of-rdp-per-country-2014-20_en.pdf.

[36] European Network for Rural Development (2013). Progress Snapshot: Measure 323 – Conservation and upgrading of the rural heritage. https://enrd.ec.europa.eu/sites/enrd/files/assets/pdf/measure-information-sheets/2014-06-19/C_Infosheet_323.pdf

[37] European Network for Rural Development (2017). Priority & Focus Area Summaries. https://enrd.ec.europa.eu/policy-in-action/rural-development-policy-figures/priority-focus-area-summaries_en

[38] European Commission (2015). Sweden's Rural Development Programme for 2014-2020 approved by European Commission. https://ec.europa.eu/info/sites/info/files/food-farming-fisheries/key_policies/documents/rdp-sweden-press-summary-26-05-2015_en.pdf

[39] European Commission (2020). Factsheet on 2014-2020 Rural Development Programme for Sweden. https://ec.europa.eu/info/sites/info/files/food-farming-fisheries/key_policies/documents/rdp-factsheet-sweden_en.pdf

[40] European Commission (Undated). Rural development programmes 2014-2020. https://ec.europa.eu/info/sites/info/files/food-farming-fisheries/key_policies/documents/rdp-2014-20-list_en.pdf
