# Supplementary material for: A harmonized and spatially explicit dataset from 16 million payments from the European Union's Common Agricultural Policy for 2015
Source: Patterns (N Y). 2021 Apr 9;2(4):100236. doi: 10.1016/j.patter.2021.100236 (PMC8085596; doi:10.1016/j.patter.2021.100236)
Supplement: Document S3. Article plus supplemental information [file mmc3.pdf]

Descriptor

Patterns

A harmonized and spatially explicit dataset from 16 million payments from the European Union's Common Agricultural Policy for 2015

Graphical abstract

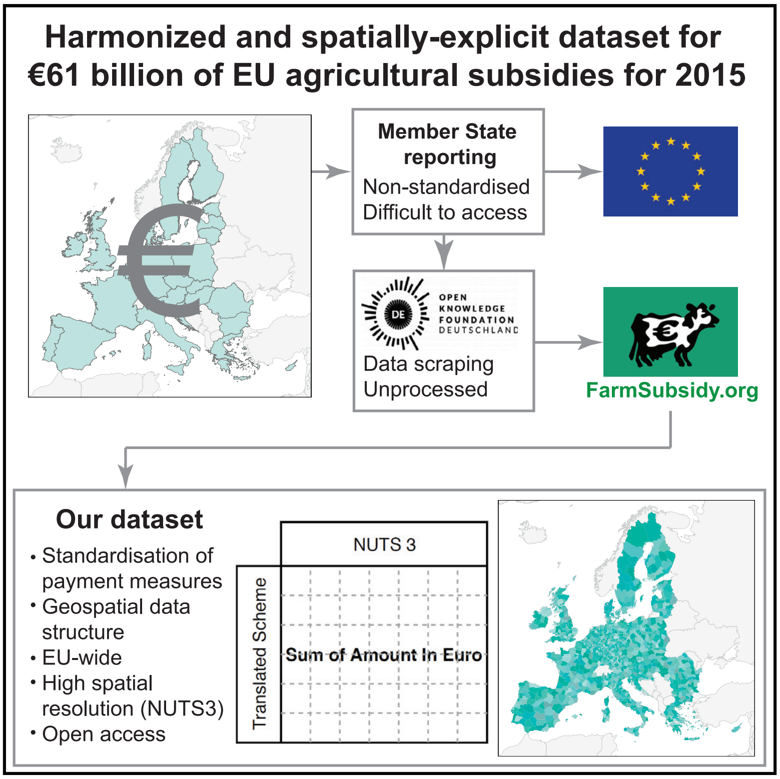

Highlights

- New dataset of €61 billion in public spending under the EU's largest budget item
- Purpose and location mapped from 16 million individual payments in Europe from 2015
- Enables both detailed and aggregated analysis of agricultural spending
- Dataset well validated and can be combined with outcome data to assess policy effect

Authors

Kimberly A. Nicholas, Frida Villemoes, Edmund Aristid Lehsten, Mark V. Brady, Murray W. Scown

Correspondence

kimberly.nicholas@lucsus.lu.se

In brief

Public spending under the European Union's Common Agricultural Policy has been difficult to analyze at a fine spatial resolution. Here we present a dataset that for the first time harmonizes spending across member states by location and purpose drawn from 16 million individual payments in 2015. When coupled with existing data on social and environmental outcomes, our spending data enable analysis of how effectively public money is being spent to achieve policy goals.

Nicholas et al., 2021, Patterns 2, 100236
April 9, 2021 © 2021 The Authors.
https://doi.org/10.1016/j.patter.2021.100236

CellPress

Patterns

CellPress
OPEN ACCESS

Descriptor

A harmonized and spatially explicit dataset from 16 million payments from the European Union's Common Agricultural Policy for 2015

Kimberly A. Nicholas,[1,5,*] Frida Villemoes,[1] Edmund Aristid Lehsten,[1] Mark V. Brady,[2,3] and Murray W. Scown[1,4]
[1]Lund University Centre for Sustainability Studies (LUCSUS), Box 170, 22100 Lund, Sweden
[2]Agrifood Economics Centre, Department of Economics, Swedish University of Agricultural Sciences (SLU), 22070 Lund, Sweden
[3]Centre for Environmental and Climate Science (CEC), Lund University, 22362 Lund, Sweden
[4]Copernicus Institute of Sustainable Development, Utrecht University, 3584 CB Utrecht, the Netherlands
[5]Lead contact
*Correspondence: kimberly.nicholas@lucsus.lu.se
https://doi.org/10.1016/j.patter.2021.100236

THE BIGGER PICTURE Transparency is a key principle of the European Union (EU), but previous spending on the EU's largest budget item, farm payments under the Common Agricultural Policy, has been difficult to analyze and compare at the EU level. We have created the first dataset that makes it possible to map and analyze farm payment spending by location and purpose across all member states. We hope that these data will be used to assess the effectiveness of the Common Agricultural Policy, to highlight areas where public spending is most supporting public benefits, as well as areas where spending could be redirected to more effectively support the stated goals of the policy. Sustainable agriculture is essential to meet human needs while meeting social goals, including European Commission climate and biodiversity targets. Our dataset can help to evaluate the role agricultural policy is currently playing and identify key areas where public support is needed to achieve these goals.

1 2 3 4 5 **Development/Pre-production:** Data science output has been rolled out/validated across multiple domains/problems

SUMMARY

The Common Agricultural Policy (CAP) is the largest budget item in the European Union, but varied data reporting hampers holistic analysis. Here we have assembled the first dataset to our knowledge to report individual CAP payments by standardized CAP funding measures and geolocation. We created this dataset by translating, geolocating to the county or province (NUTS3) level, and consistently harmonizing payment measures for over 16 million payments from 2015, originally reported by EU member states and compiled by the Open Knowledge Foundation Germany. This dataset and code allow in-depth analysis of over €60 billion in public spending by purpose and location for the first time, which enables both individual payment tracing and analysis by aggregation. These data are representative of the distribution of annual CAP payments from 2014 to 2020 and are of interest to researchers, policy makers, non-governmental organizations, and journalists for evaluating the distribution and impacts of CAP spending.

INTRODUCTION

Agriculture provides essential food and livelihoods for people, but land-use change, primarily driven by agriculture, also causes the majority of global biodiversity loss[1] and 23% of climate heating.[2] The current food system is criticized for harming both planetary and personal health, recognizing the urgent need to transform to healthy and sustainable food systems.[3,4] Agricultural subsidies globally total over $700 billion (€640 billion),[5] with many reinforcing harmful practices.

The European Union (EU) has pledged to be a global leader in sustainable agriculture, including making the "farm to fork" sustainable agriculture strategy a cornerstone of the European Green Deal.[6] Currently the principal policy for European agriculture is the Common Agricultural Policy (CAP), the largest budget item in the EU. The CAP consists of two pillars: Pillar I comprises

This is an open access article under the CC BY license (http://creativecommons.org/licenses/by/4.0/).

about 71% of CAP spending as direct payments to farmers and 4% to market measures, with the remaining 25% of funding supporting Pillar II programs in rural development and environmental measures.[7]

The overriding aims of the CAP are to support farmers' income, improve agricultural productivity and competitiveness, ensure a stable supply of affordable food, and support rural development, climate action, and sustainable resource management.[8,9]

However, the CAP has faced wide-ranging criticism, including for increasing income inequalities and for underresourcing goals for rural development and environmental protection by overfinancing ineffective income support.[10,11] The CAP is currently under reform for 2021–2027.[12] The European Commission has communicated that the future CAP should evolve in line with the Sustainable Development Goals.[13]

EU member states are obligated to report spending to comply with the EU's principle of transparency, including regulations with specific obligations for publishing CAP payment recipients.[8] Specifically, in Article 111 of Regulation (EU) No. 1306/2013,[14] member states are required to report the following information on a single website for at least 2 years following publication: payment beneficiaries (first and last names of individuals or full legal name of associations or companies), the municipality where the beneficiary is registered (and postal code "where available"), the amounts of payment corresponding to each measure, and "the nature and description of the measures" for both EU and member state contributions. The European Commission maintains a web page[15] with links to each country's CAP payments reporting website, where they state, "To ensure full transparency, EU countries publish information relating to the beneficiaries of all common agricultural policy (CAP) payments on their national websites" (see supplemental information). Currently, all farms or farmers have an individual ID number, but the system depends on the individual member state, and there is no common system in the EU (R. Hießerich, Federal Ministry of Food and Agriculture Germany, personal communication, May 8, 2020).

In practice, it is currently extremely difficult to get an overview of CAP spending at a finer level than the national summaries published by member states or aggregated EU analyses published by the European Commission, because data are fragmented and incomplete. Each member state maintains its own database for reporting CAP spending, each of which uses a different format and includes different information. Data access is a problem; most of these transparency portals allow only specific searches (it is not possible to see or download all the data without writing your own code to do so), and most portals remove data older than the latest 2 years.

Crucially, there is no universal standard for the "nature and description of measures" that member states are required to report, so there has been no way to harmonize the data (by which we mean standardize payments so that their purpose, recipient, and location can be compared and aggregated between member states). Such harmonization is needed to gain a comprehensive overview of where CAP spending went and for what purpose, as well as to combine the CAP data with other datasets, for example, on environmental and social outcomes that the CAP is intended to promote, to assess the policy's effectiveness in practice.

The goal of the present study is to develop and present the first spatially explicit database of CAP spending, harmonized across measures and member states for the fiscal year 2015. To do so, we created a "Rosetta Stone" to align measure names reported between countries (called "scheme" in the raw data and our code) to a standardized list. This spending averages €58.2 billion annually over the 2014–2020 program period.[7] The raw payments data were originally reported by EU member states, and scraped from 27 different reporting websites by the Open Knowledge Foundation Germany. They average over 600,000 records each (range: 8,600 records reported for Malta to 3,235,524 records reported for Romania), where each line represents a payout amount to a given recipient under a given measure. We performed language translation and aligned a given scheme name with the purpose of the measure by using machine translation and native speakers, consultation with local agricultural experts, and extensive data formatting and processing. The resulting database enables analysis of the purpose and location of CAP spending for the first time and facilitates future analysis of the social and environmental benefits of this spending, for example, in relation to CAP and sustainability goals.

RESULTS

Creating the harmonized payments database

In brief, the workflow proceeded in two stages using a Python script (Figure 1). First, we processed raw data files reporting CAP payments for each country from 2015 (obtained from farmsubsidy.org) to create a "translated" version of the country file. The translated version included additional columns appended to assign each row to a standardized measure name by using the Rosetta Stone we created as a lookup table, amount of spending in euros, and a NUTS3 region (in the EU's Nomenclature of Territorial Units for Statistical Analysis) for spatial analysis. These translated data files are suitable for individual country analysis or detailed analysis of particular measures across the EU. Second, we aggregated all of the translated country files to produce the "condensed year" file, which contains the total amount of spending for each CAP measure and NUTS3 region in the EU, suitable for broader-scale analyses and aggregation. Code to reproduce the full dataset is available on GitHub.

We used raw CAP payment data scraped from country websites by FarmSubsidy.org, which is a project of the Open Knowledge Foundation Germany, a non-profit organization working on transparency of public money. The Farmsubsidy.org project is currently unfunded and maintained by volunteers,[16] with code released under open license with the intention to be maintained by the community.[17] They publish the data exactly as published by national governments.[16]

We downloaded the raw CAP payment data for all available member states and years from FarmSubsidy.org on July 15, 2019, using the Linux command $wget -r https://data.farmsubsidy.org/latest/ (see instructions for download and for setting up the file structure in the readme file on our GitHub, https://github.com/kanicholas/CAP-farm-payments). Note that files are compressed and need to be extracted; we used Archive Manager on Ubuntu.

There were wide variations in the raw CAP payments data reported by member states (summarized in Table S1). Here we

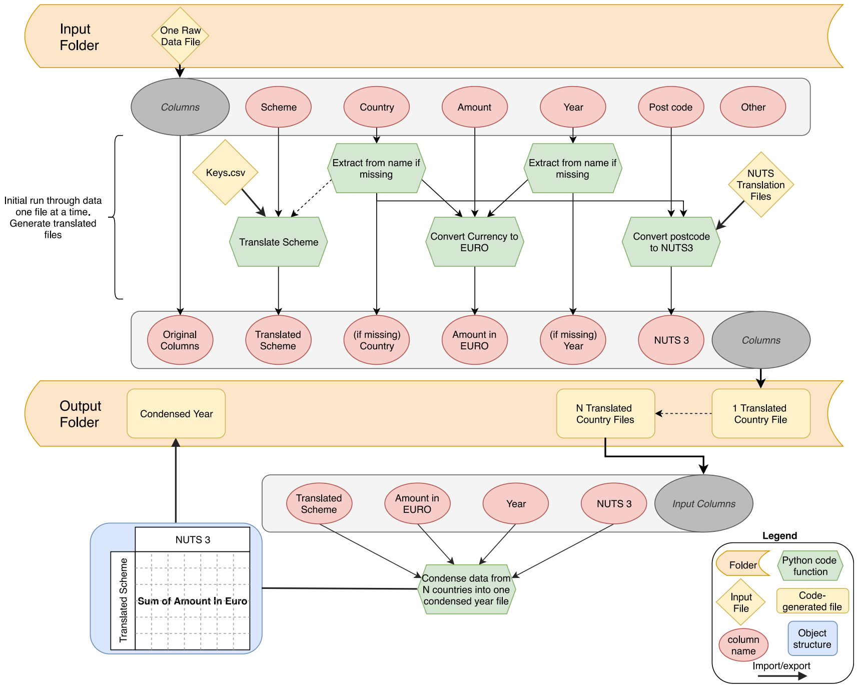

Figure 1. Workflow for generating the data for CAP spending by NUTS3 region within the EU and one of 102 CAP payment measures

Input files include raw data from farmsubsidy.org; the keys-csv translation file to align country-specific measure names with standardized measure numbers, generated by the research team (this is the "Rosetta Stone" file, Table S2, with meta-data about measure names removed); and files to translate postal codes to NUTS regions (downloaded from EU). Steps in the Python code are shown in green. The first phase takes in raw country data from farmsubsidy.org and outputs the translated file, where rows are matched to NUTS3 regions and CAP measures. The second phase extracts the relevant columns from each country file, and for all countries within 1 year produces a condensed file of CAP spending by measure per NUTS3. See code and readme file on our GitHub.

provide an overview of the assumptions we made to harmonize the data between countries (see supplemental information for full details).

We built our database using data from 2015; where data from 2015 were not available or were incomplete, we used the closest available year (2014 for Denmark and 2016 for Bulgaria, Sweden, and the Czech Republic). Although most member states list the payment year in their raw data, which was repeated in the file name, they do not clarify how years are reported. The CAP financial year runs from October 16 to October 15, with the payments published the following year. We assume that data files stating a year of 2015 refer to the majority year the payment was made (as stated by Germany on their transparency website, where searching 2018 is stated to apply to payments made from October 16, 2017, to October 15, 2018).[18] Thus, we believe the majority of our data report spending undertaken from October 2014 to October 2015 and reported in the spring of 2016. We attempted to match all payments listed to the standard list of EU measures and converted all payments to euros. We classified about 1% of payments as from national rather than European funding (see supplemental information).

We used a combination of postal codes matched to NUTS3 regions and manual matching to obtain a standardized geolocation for each payment entry. In total, we successfully geolocated 83% of the payments in our database. We could not geolocate payments for nine countries where postal codes were not provided in the raw data, nor for about 19% of payments in Sweden that do not follow NUTS3 borders. In total we were unable to geolocate about €9 billion in total payments beyond the national level (about 15% of total payments in our dataset). In addition, we were unable to match 2% of reported locations to postal codes. This 17% of the dataset is thus geolocated to the national, NUTS0 level. To harmonize measure names across countries, we created a master Rosetta Stone file (Table S2) where we aligned names for the CAP payment measures given by member states in their national language to a common English language standard label. For the standard label, we used the "Description of Measures" published by DG AGRI, sometimes cited as Ares (2018),[19] which lists 102 individual measures (27 in Pillar I and 75 in Pillar II), drawn from 10 different pieces of underlying regulations (listed in Table S3). Meta-data on the structure of the Rosetta Stone are given in Table S4.

Fourteen countries reported a standard measure identifier, such as the Roman numeral for the measure name in Ares (2018), that made matching measure names straightforward (see Table S1). For the remaining 13 countries, matching measure names required a combination of machine translation and native speaker assistance, research on national agency websites, and direct contact with national offices and country experts. Where judgment was required to match a reported measure name with the appropriate standard label, we developed a classification system for assessing the certainty of our match. We used the full dataset (all levels of match certainty) for analysis, but report the certainty level of matches by country and measure name for most of the measures in Table S5 in case others wish to have a more stringent cutoff. Four of the regulations underlying the 102 measures expired during the 2014–2020 CAP period, but remained valid for payments through 2015,[20] so member states used a mix of old and new terminology in reporting the payments in our dataset. See Table S6 and notes in the Rosetta Stone regarding additional possible matches where short or ambiguous wording was reported for measure names.

Data records

We have created two sets of data records using the methods described above, both of which are structured to reside in the "Output" folder on our GitHub (see Figure 1). First, we have created a "Translated country file" for each of the raw country data files. This maintains the original raw data for each country

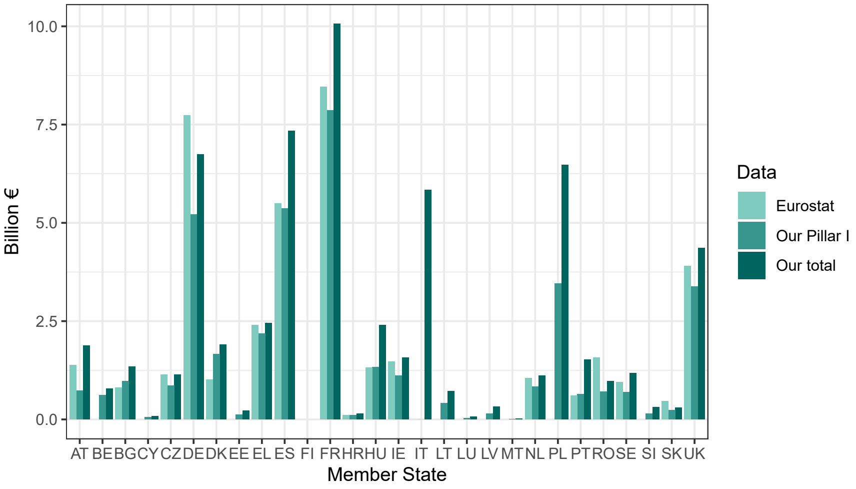

Figure 2. Comparison of farm payments for production reported by Eurostat with the payments in our database

Eurostat payments reported are shown in light green, with our payments shown in both medium green (Pillar I) and dark green (total payments, Pillar I + Pillar II). Payments are for the years reported in Table S1 (2015 except for four countries.)

(every payment reported in the raw transparency data, e.g., over 3 million records for Poland, including any personal identifying information of recipients where such data were originally reported by member states) and appends to it additional columns to facilitate analysis, including standardizing to translated measure name, adding country and year (if missing), converting currency to euros, and adding the NUTS3 region (Figure 1).

These 27 translated country data records were then further processed to create the condensed year data record, which shows the payment amounts spent on each CAP measure for each NUTS3 region for the given year. For convenience, the resulting condensed files from running the workflow described in Figure 1 are provided in Tables S7–S9 for 2014, 2015, and 2016, respectively. These three files are themselves the input to the technical validation, as described below.

Technical validation

The factors in public data reporting and curation that made this dataset difficult to generate also made it difficult to validate, namely, the impermanent availability of the raw data; the lack of coherent, centralized reporting that covers all CAP spending; the tendency to report total funding received during the 7-year CAP period rather than by year; the failure to consistently distinguish between EU and national funding in reporting payments; and the lack of accessible data showing payments by measure or by location finer than member state. Based on the current state of public data reporting on CAP spending, described below, we believe we have used the best available validation data, but we were not able to identify a publicly available source of information against which to comprehensively validate our data. Nonetheless, based on our validation efforts we are confident that these data represent the best currently publicly available data on CAP spending across the EU. We hope that the publication of our dataset spurs greater inquiry and transparency for the member states and the EU to report these data in a directly usable format (broken down by year, unique CAP measure ID, measure name, and location, including postal code), as we detail in the recommendations below.

Original data validation

Because the transparency legislation requires data to be available for only 2 years, it was no longer possible to download the original data from the member states for 2015 for validation; we thus rely on the accuracy of the data scraped and stored by Farmsubsidy.org. Please see R code on the GitHub to read in the translated and condensed files (allocating payments to NUTS3 regions by CAP measure for all member states, using the years noted above) and perform the validation analysis and produce the figures and tables described in this section.

Reporting of CAP spending across the EU

The EU reports annual spending in its expenditure and revenue data under Section 2, "Sustainable Growth: Natural Resources."[21] Spending is reported in the broad category of either Pillar I (European Agricultural Guarantee Fund [EAGF], line item 2.0.1) or Pillar II (European Agricultural Fund for Rural Development [EAFRD], line item 2.0.2), but a finer breakdown by the 102 measures under these broad categories is not available.

A broad comparison between our data and EU reported spending at the pillar level confirms very close agreement. Of the 90.5% of payments we were able to attribute to measures, our data total €38.9 billion for Pillar I and €16.0 billion for Pillar II for the years used (centered around 2015, with four countries using data from 2014 or 2016 as noted above). For the same countries and years, Eurostat reports €40.0 billion spending in Pillar I and the Commission reports €15.6 billion in spending for Pillar II. These totals are broadly in line with the budget and spending during the 2014–2020 CAP period. In our dataset, we were able to identify only a handful of payments made under measures from national as opposed to EU funding (totaling €0.62 billion, roughly 1% of our payment total) (Table S10), which could partly explain the difference between our total and that from the Commission.

Eurostat administers data on "subsidies on production" (item code 25000) in their "Economic accounts for agriculture by NUTS2 regions" (Table S11). However, after repeated requests for Eurostat support during 2018 and 2019, it remained unclear to us what these data actually represented in relation to CAP spending. The Eurostat "subsidies on production" total just over €47 billion for 2015 at the NUTS0 (member state) level, but many NUTS2 regions and even several member states contain no data in this table.

We compared the payments reported by Eurostat for each member state with our data, finding generally good agreement, although the payments in our dataset were generally slightly higher than those reported by Eurostat, with the exception of Denmark (Figure 2; Table S12). Our data generally show the expected pattern that Pillar I comprises the majority (approximately

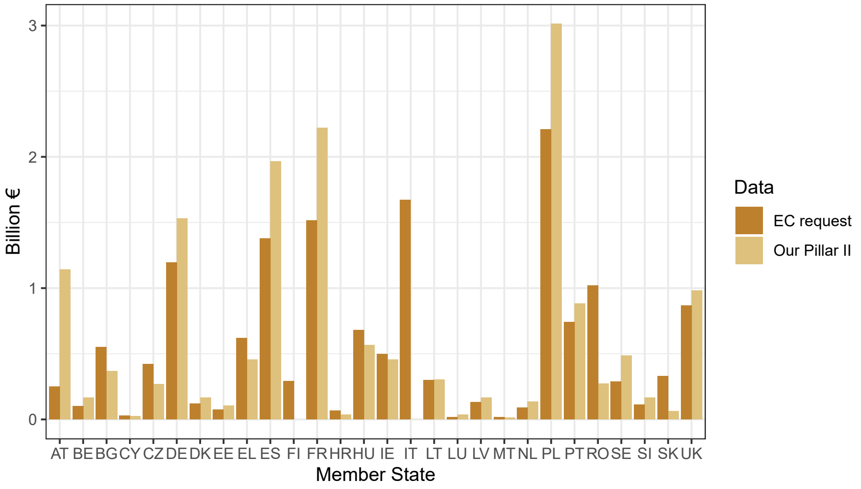

Figure 3. Comparison of Pillar II payments from 2015 reported by the European Commission and the Pillar II payments in our database

Data from the Commission were obtained upon request from the ESIF Open Data Platform (see text and Table S10). Note that European Commission data are all from 2015, and our data are for the years reported in Table S1 (2015 except for four countries.)

and to regions in the second (17% of payments could be geolocated only to the national level).

We analyzed agreement between our data for Pillar II spending and the data sent by the Commission for a set of 832 specific measures within countries, finding generally good agreement (most points lie close to the one-to-one line in Figure 4; Table S13). All points would be on the one-to-one line in Figure 4 if there were perfect agreement between our dataset and that of the European Commission. The gray lowess line indicates that on average, European Commission payments are reported as slightly higher than our data, especially as spending recorded by the Commission increases. This trend is caused largely by some of our data containing zeros or missing data where the Commission has recorded spending.

Taken together, the results of this analysis indicate that our independently collected and transparently constructed dataset is reasonably close to the data used by the European Commission, although not complete for all measures in all countries. However, there are some specific countries where Pillar II measures in our dataset are either substantially more (above the 1:1 line, such as SI.V/B.3.4) or substantially less (below the 1:1 line, e.g., LU.IV/A.18) than reported by the Commission. Although small in the overall CAP budget, if undertaking detailed analyses at the country or measure level, one should scrutinize such payments with caution.

At the NUTS2 level, we were able to compare total CAP payments for 148 NUTS2 regions that were shared between our data and Eurostat. We found generally good agreement, with most points lying close to the one-to-one line, especially at higher payment values, where the gray lowess line converges with the one-to-one line (Figure 5A). This agreement was reinforced by the analysis of the rank order of these 148 NUTS2 regions between our data and Eurostat, showing close overlap (Figure 5B).

three-quarters or more) of the total CAP spending, with the notable exceptions of Austria, Hungary, and Poland, where about half of total spending in our data came from Pillar II.

Reporting of CAP spending by member states

Distinguishing the purpose for each measure of reported funding is particularly difficult for Pillar II at the member state level, because reporting is spread out over many different venues, often aggregated across a 7-year CAP period rather than broken down annually, and often national funding sources are not clearly distinguished from EU funding (see supplemental information for more details).

The four member states who receive the most funding from Pillar II, EAFRD funding across the 7 years of the 2014–2020 CAP, are France (€11.4 billion), Italy (€10.4 billion), Germany (€9.4 billion), and Poland (€8.7 billion).[22] Thus, these member states are especially relevant for analysis, and their reporting quality is of particular concern for clarification and improvement.

Overall agreement for expenditure by measure and member state under Pillar II was generally reasonably close between our data and the data obtained upon request from the European Commission via the ESIF Open Data Platform (personal communication, May 14, 2020) (Figure 3). However, our data were notably higher than that from the Commission for Austria, Spain, France, and Poland (Figure 3). Here the Commission reported Pillar II spending in Italy of about €1.7 billion, which would fit reasonably well, as about 30% of our total of €5.8 billion (which we were unable to analyze by measure, as the raw data reported only the measure name ''Total''). Note that data for this figure came only from 2015 from the Commission, but from 2014 or 2016 for four countries in our dataset as noted above.

From comparing our data with the most detailed data available from official sources, we find generally good agreement, with our data overall showing slightly lower payment values than reported by the European Commission broken down by measure in Pillar II, and slightly higher values than reported by Eurostat for all measures at the NUTS2 level. This is consistent with our inability to successfully match all payments to measures in the first case (9.5% of our payment data remained unmatched to measure)

Missing data and uncertainties

Overall, we were able to successfully match most payments from most countries both to the measure they supported (90.5% of payments) and to their spatially explicit geographic location within a country (83% of payments; most of the unmatched were in countries that did not report postal codes or other location information). Note that the vast majority of countries where measures were listed were able to be matched, as Italy listed no measures and comprised nearly 10% of the total payments in our database. See Python code ''error_percentages.py'' for

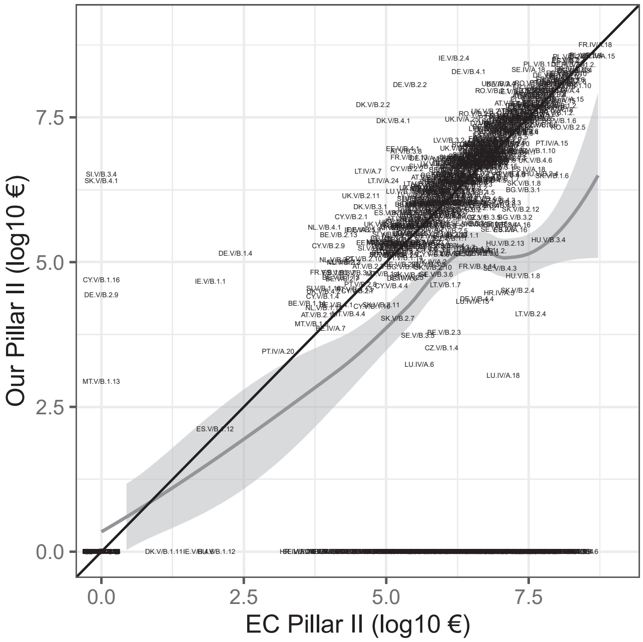

Figure 4. Comparison of Pillar II payments from 2015 reported by the European Commission and the Pillar II payments in our database, by measure and member state

Data from the European Commission (EC) were obtained upon request from the ESIF Open Data Platform (see text and Table S10). Each of the 832 data points represents a unique PII measure in each country (see Table S13 for 55 negative values that were not plotted due to log transform scale). The black line is a one-to-one line; all points would lie on this line if the two datasets were identical. Points above this line are where our dataset reported higher values than the EC, and points below this line are where the EC data were higher. The gray line is a lowess curve, with 95% confidence interval shaded in light gray, indicating that overall, the EC data reported higher payment values than our data. The EC data also report spending under some measures in some countries that were missing or zero in our dataset (shown as values along y = 0). Note that EC data are all from 2015, and our data are for the years reported in Table S1 (2015 except for four countries.)

the euro amount of payments unmatched, and ''error_list.py'' for the names of reported measures unmatched, on our GitHub. A country-level breakdown of the percentage of total funding within the year studied matched to both measure and location is shown in Table S1.

For many countries, less than 1% of payments were unmatched to either a measure or a location. More than 98% of all payments within a country were successfully matched to a measure, except in Greece (7.9% of payments unmatched to measure, due to high payments to six measure names not matched to the master list), Latvia (4% unmatched), and Denmark (4% unmatched to measure, due to errors in reported measure names); see Table S1. Other than the nine countries who did not report postal codes and were therefore 100% unmatched to a NUTS3 region, the only countries with over 2% of payments unmatched to location were Sweden (19.1% unmatched to NUTS3, due to non-overlap between postal codes and NUTS3 regions in Sweden); the Netherlands (13.4%), France (4.7%), Slovakia (4.4%), Italy (4.0%), and Malta (3.6%).

A number of countries had specific errors or issues with their measure formatting that required special processing or analysis. In brief, common errors included double entry of both total payments and subtotals for the same recipient (Latvia); ambiguous entries, such as article numbers that could apply to multiple measures (Denmark); reporting of old/expired measures under the previous CAP (e.g., Estonia); measure names not reported at all (Italy); and repeated entries for the same measure name, with variations in punctuation or spelling (e.g., Denmark, Romania), among others. See details about how country-specific issues were handled in the Python code and in Table S1, column ''Errors and uncertainties in raw data.'' This column could be used to inform member states of key areas to focus on for improvement in reporting.

Although we believe this dataset represents a substantial step forward in transparency of EU budget spending, data users should take care to understand the assumptions made. In particular, as described above and in the supplemental information, areas for attention include distinguishing national versus EU measures and the certainty of measure matches to the master list due to ambiguity in the raw data. Ambiguity in matching measures to the master list could arise both from how member states reported measures and from the existence of multiple measures dealing with the same topic. For example, a member state reporting the measure name ''Advisory services'' could apply to at least three measures; see the supplemental information. Further, there exist multiple measures dealing with the same topic (see Table S6).

For detailed analyses of specific measures, users should consult the ''Notes'' column by country in the Rosetta Stone (Table S2) and the match certainty rating in Table S5. Within Pillar II, given the overlapping intents and names between measures, it is probably most accurate to combine payments within similar measures for analysis by broad purpose (e.g., using Table S6), rather than focusing in detail on individual measures.

DISCUSSION

Potential reuse value

We have undertaken to harmonize existing data reported by individual member states on public spending on the CAP, which have previously been very difficult to access in a way that facilitates the analysis and comparison essential for transparency. These data are important and relevant for researchers; policy makers; non-governmental organizations working with the Sustainable Development Goals, environmental stewardship, and other policy goals; and journalists reporting on public spending and government oversight, as well as the EU member states themselves and their citizens.

Given the high public interest in these data as the CAP reform discussions are ongoing, as well as extensive and ongoing calls for increased transparency of CAP spending, we believe the reuse potential for these data is high. This is especially the case when competing priorities highlight the need to use public resources wisely in pursuing urgent social goals such as sustainable food production, rapidly reduced climate pollution and enhanced natural carbon sinks, and biodiversity conservation. Despite the huge amounts of CAP spending, lack of suitable data at the appropriate time and scale is hindering effective evaluation of CAP measures in relation to their goals.[23]

We hope that this data harmonization effort can be carried forward to support more transparent and harmonized reporting by

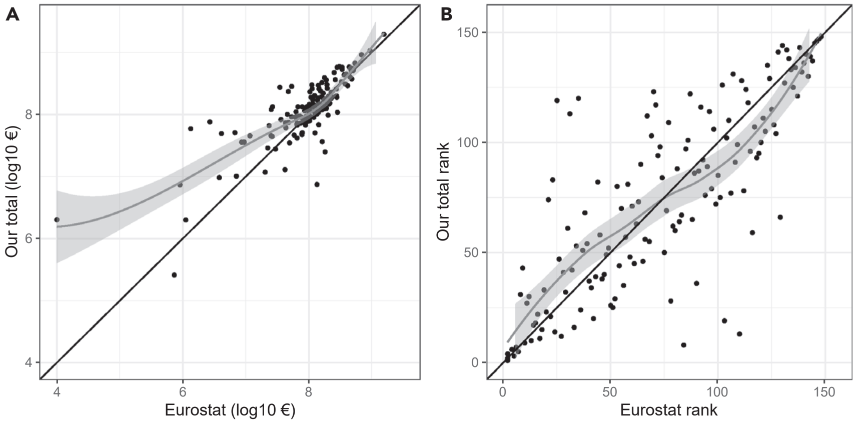

Figure 5. Comparison of CAP payments between Eurostat and our data at the NUTS2 level

Comparison of CAP payments from 148 NUTS2 regions that were common between our data and those of Eurostat. The black line is a one-to-one line; all points would lie on this line if the two datasets were identical. Points above this line are where our dataset reported higher values than Eurostat, and points below this line are where the Eurostat data were higher. Note that three data points were excluded from (A), with zeros for Eurostat. Also note that Italy NUTS2 regions were all excluded with all zeros in Eurostat. The gray line is a lowess curve, with 95% confidence interval shaded in light gray. Its position above the one-to-one line indicates that for a few NUTS2 regions, our dataset was higher than that of Eurostat, but the two converged closely at higher payment values. (B) displays the rank order of the NUTS2 regions; the close agreement between the one-to-one and the lowess lines indicates a good agreement.

member states in the coming CAP spending period 2021–2027 to support ongoing analysis and collaboration toward achieving Europe's policy goals for sustainable agriculture.

We also see high potential to conduct further analyses with existing spatial data, now that these CAP payments have been made spatially explicit for the first time. For example, it would be interesting to analyze trends in social and environmental agricultural indicators related to the goals of the CAP, compared with payments made. (See Scown et al., 2020, for an analysis of these CAP payments compared with income, greenhouse gas emissions, and high-nature-value farmland location.)[11] We note that the CAP dataset documented here can be analyzed in conjunction with a previously published dataset of 127 variables relevant for agriculture and the Sustainable Development Goals in Europe, such as greenhouse gas emissions from agriculture, water abstraction, and rural risk of poverty.[24]

Recommendations

The difficulty we encountered in creating this harmonized dataset, and the remaining gaps and uncertainties in the data, demonstrates the need for common-sense reforms to streamline CAP payment reporting and data curation. Here we echo previous calls for EU member states to release data "according to a common format so that it is possible to analyse the data in a meaningful way across the European Union."[16]

Below we offer recommendations to make the CAP payment data reported by member states more standardized to facilitate analysis and transparency. Some of our recommendations are very basic (consistently following existing regulations and good practices in data curation), while others would require updates or changes in current practices, but would offer substantial benefits to public transparency. These recommended improvements to data reporting would enable detailed analyses of CAP spending, which could inform and improve the overall performance of the CAP. These recommendations could be implemented at various levels, for example, in guidance for member states on reporting their strategic plans under the post-2021 CAP.

Recommendations for future improvement in CAP data reporting

Most fundamentally, we recommend that future member state reporting of CAP spending require the following documentation to be reported through each country's data transparency portal:

- Member state payment reporting should include a column with the standardized EU-wide measure name and associated unique identifier for each measure, alongside the member state name used for the measure name to minimize errors. Further, member state reporting should include a column noting funding source for payments made (e.g., distinguishing payments made from the EAGF for Pillar I, from the EAFRD for Pillar II, and from national sources).
- A dictionary file that lists the measure names reported in the native language/format, referenced to a standard list of measure names and unique identifiers provided by DG Agri, should be included. The Rosetta Stone document provided in this paper (Table S2) could serve as an initial template to do so, although it would need to be updated for the measures adopted in the 2021–2027 CAP.
- A document listing the names of and briefly describing any additional national measures reported in the payment data (those not included in the standardized EU measures) should be included.
- The name of the agency and department responsible for curating the payment data, as well as a contact person, should be clearly specified, and their contact information for questions about the dataset should be provided.
- Meta-data should be provided for the whole dataset, including an explanation of negative values.

To facilitate analysis and minimize errors, the *format of data reported* should always include the following elements and consistently follow data curation conventions:

- In addition to any data preferred to report by the reporting member state, CAP reporting should always include the

following data: country name, year, standardized EU measure name and identifier, recipient identifier (name *or* recipient ID, with unique European identifier for legal entities), recipient postal code, payment amount, currency used to report payment amount (if data were collected in a currency other than euros and converted to euros, the exchange rates used to convert to euros should be reported in the meta-data).

- A column to distinguish the source of payments made (EU or member state funds and their proportional contributions) should be included.
- Identifying data (measure name, ID, recipient name, etc.) should be completely filled in for each and every row of payments reported (not left blank under headings assumed to be carried down until a new entry appears, which hinders analysis). Where values are zero or not applicable, appropriate codes should be used and noted in the meta-data.
- Measure names and other text responses should be reported from a standardized list or pull-down menu, not entered by hand, to reduce the frequency of duplicate and erroneous measure names.
- When numbering is used as part of measure names, it should be done using unique consecutive numbers (such as 001, 002, …). Sometimes current numbering conventions yield non-sequential sort orders, such as measures in Denmark that mix numbers with names to label their measures; sorting them yields 1, 10, 11, 12, … 2, 20, 21, 22, ….
- Validation should be carried out by the reporting agency responsible before reporting data, to minimize errors. That is, the data should be totaled and reconciled with official statistics to ensure all payments have been accounted for, and standardized, unique measure names and ID numbers should be used to ensure every payment reported can be uniquely associated with its recipient and purpose, and to avoid errors in measure names.

Additional suggestions to improve data reporting (beyond current mandates) are as follows:

- Payment data would be much more usable in spatial format if postal codes were required to be reported (current regulation requires postal codes to be reported "where available"). Because they encode geospatial information, postal codes are much easier to link to open-source spatial databases that allow meaningful analysis than the currently used geospatial identifier, municipality.
- Member state reports would be much more useful if they directly reported NUTS3 region (and stated which NUTS3 version was used in the meta-data), since the documents available from Eurostat did not always convert postal codes to NUTS3 regions with high accuracy. This would enable detailed spatial analyses of CAP payments against other agricultural statistical information, for example, from Eurostat.
- Member states should make it possible not just to search by specific criteria, but also to directly download the full year's payment data from their websites (as a few countries have already done).
- We recommend implementing a unique European identifier for legal entities to help match recipients across countries and avoid duplicate entries. (The Open Knowledge Foundation Germany notes that the US and Mexican governments publish unique recipient ID codes that allow tracking the same recipient over different years and different datasets.)[16] A unique identifier would also help address privacy concerns.

New suggestions for reporting administration

Current legislation requires each member state to report its payment data on its own websites. If member states follow the recommendations above, their reported data will be much more usable. However, stronger guidance and coordination at the EU level would make the data much easier to analyze than downloading it from 27 separate websites. At a minimum, as noted above, the appropriate EU agency, such as DG AGRI, should produce a spreadsheet template with standardized measure names against which each member state should submit a dictionary file mapping how its reported measures map to the master measure names (essentially making the Rosetta Stone that we have created from scratch here the standardized reporting framework). Centralized curation of the data would likely improve accuracy and accessibility and make evaluation against result and impact indicators possible. It would also facilitate analysis if the EU made geospatial data on postal codes (.shp files) open source (they are currently proprietary). It would be a great help if centrally reported data were available in data (spreadsheet) format by year and pillar (instead of 118 separate PDF files for rural development programs across 2014–2020).

Future research

For further analysis of past CAP spending, it would be helpful to expand the work done here to align measures reported during the entire 2014–2020 CAP (beyond 2015) with the standardized measure list. However, acquiring historical CAP spending data from member states is a challenge, since they are required to make the data available for only 2 years, after which most countries seem to remove older data from their transparency websites. Some additional historical data on country-level CAP payments are available from Farmsubsidy.org, although they are not able to archive all years given their current all-volunteer status. With additional resources, further research could be done to scrape and archive the data reported by member states for the current reporting period. Going forward to the CAP starting in 2021, we urge the European Commission and member states to follow the recommendations above so that such extensive compilation and harmonization will not be necessary to reveal how public money is being spent.

EXPERIMENTAL PROCEDURES

Resource availability

Lead contact

Further information and requests for resources and reagents should be directed to and will be fulfilled by the lead contact, Kimberly Nicholas (kimberly.nicholas@lucsus.lu.se).

CellPress
OPEN ACCESS

Materials availability

Note that the full raw data files for CAP payments were originally published according to EU transparency law by member states and archived by the Open Knowledge Foundation Germany (https://data.farmsubsidy.org/latest/), and the code begins with extracting the raw files from that archive.

Data and code availability

Our Python and R scripts, instructions for accessing the raw data, and associated output data files are available on GitHub at https://github.com/kanicholas/CAP-farm-payments.

SUPPLEMENTAL INFORMATION

Supplemental information can be found online at https://doi.org/10.1016/j.patter.2021.100236.

ACKNOWLEDGMENTS

This research would not have been possible without the hard work of many to assemble the CAP and European agricultural data. We are very grateful to the Open Knowledge Foundation for years of work in obtaining and curating the raw CAP spending data by member state, which we harmonized, standardized, and made spatially explicit here. Thank you to Stefan Wehrmeyer of the Open Knowledge Foundation for helpful correspondence.

We thank our translators Lina Vaitkunaite (Lithuanian), Markus Vihma (Estonian), Katalin Lakatos (Hungarian), Ruxandra Popovici (Romanian), Klara Winkler (German), Kristina Cekanovic (Croatian), Ivaylo Hlebarov (Bulgarian), and Juris Treibergs (Latvian) for assistance with language translations of country measures and Xoco Shinbrot and Kira Treibergs for help via Twitter to identify a Latvian speaker.

Kind assistance to match reported payments to CAP measures in national languages was provided by Jan Holub and Jana Ludvíková of the International Relations Unit at the State Agricultural Intervention Fund of the Czech Republic; Jens te Kampe of the Federal Office for Agriculture and Food, Germany; Ilze Palde of the Information and Statistics Division of the Information Department, Rural Support Service, Latvia; Giovanni Belloni at the Twitter account for the Netherlands Enterprise Agency (@RVO_Duurzaam) and Maaike Rensing at the Netherlands Enterprise Agency; and Liina Arumägi, Budget and Analysis Department, Agricultural Registers and Information Board of Estonia.

Thank you to Ronald Hießerich, of Unit 615, "EU-Agrarfunding, Responsible Authority" of the Federal Ministry of Food and Agriculture (BMEL), Germany, for helpful correspondence regarding payment periods, recipient IDs, and other technical aspects of CAP payment administration.

This research was supported by the Swedish Research Council (Vetenskapsrådet) grant number 2014-5899. The research presented in this paper is a contribution to the strategic research area Biodiversity and Ecosystems in a Changing Climate, BECC.

AUTHOR CONTRIBUTIONS

Conceptualization, K.A.N.; Methodology, K.A.N. and M.W.S.; Software, E.A.L. and M.W.S.; Validation, K.A.N., E.A.L., M.V.B., and M.W.S.; Formal Analysis, E.A.L., M.W.S., and K.A.N.; Investigation, F.V., E.A.L., M.W.S., and K.A.N.; Data Curation, K.A.N., F.V., E.A.L., and M.W.S.; Writing – Original Draft, K.A.N.; Writing – Review & Editing, K.A.N., F.V., E.A.L., M.V.B., and M.W.S.; Supervision, K.A.N.; Project Administration, K.A.N.; Funding Acquisition, K.A.N.

DECLARATION OF INTERESTS

The authors declare no competing interests.

Received: June 19, 2020
Revised: December 1, 2020
Accepted: March 15, 2021
Published: April 9, 2021

REFERENCES

1. Díaz, S., Settele, J., Brondízio, E., Ngo, H., Guèze, M., Agard, J., Arneth, A., Balvanera, P., Brauman, K., Butchart, S., et al. (2019). In Summary for Policymakers of the Global Assessment Report on Biodiversity and Ecosystem Services of the Intergovernmental Science-Policy Platform on Biodiversity and Ecosystem Services, S. Díaz, et al., eds. (IPBES secretariat) https://ipbes.net/sites/default/files/2020-02/ipbes_global_assessment_report_summary_for_policymakers_en.pdf.
2. IPCC (2019). Summary for policymakers. In Climate Change and Land: An IPCC Special Report on Climate Change, Desertification, Land Degradation, Sustainable Land Management, Food Security, and Greenhouse Gas Fluxes in Terrestrial Ecosystems, P.R. Shukla, et al., eds. https://www.ipcc.ch/site/assets/uploads/sites/4/2020/02/SPM_Updated-Jan20.pdf.
3. Willett, W., Rockström, J., Loken, B., Springmann, M., Lang, T., Vermeulen, S., Garnett, T., Tilman, D., DeClerck, F., Wood, A., et al. (2019). Food in the Anthropocene: the EAT–Lancet Commission on healthy diets from sustainable food systems. Lancet *393*, 447–492, https://doi.org/10.1016/S0140-6736(18)31788-4.
4. Recanati, F., Maughan, C., Pedrotti, M., Dembska, K., and Antonelli, M. (2019). Assessing the role of CAP for more sustainable and healthier food systems in Europe: a literature review. Sci. Tot Env *653*, 908–919, https://doi.org/10.1016/j.scitotenv.2018.10.377.
5. Food and Land Use Coalition (2019). Growing Better: Ten Critical Transitions to Transform Food and Land Use. https://www.foodandlandusecoalition.org/wp-content/uploads/2019/09/FOLU-GrowingBetter-GlobalReport.pdf.
6. European Commission (2019). Communication from the Commission to the European Parliament, the European Council, the Council, the European Economic and Social Committee and the Committee of the Regions (The European Green Deal). https://ec.europa.eu/info/sites/info/files/european-green-deal-communication_en.pdf.
7. European Parliament (2020). Financing of the CAP. https://www.europarl.europa.eu/factsheets/en/sheet/106/financing-of-the-cap.
8. European Commission. Key Policy Objectives of the Future CAP. https://ec.europa.eu/info/food-farming-fisheries/key-policies/common-agricultural-policy/future-cap/key-policy-objectives-future-cap.
9. European Commission. The Common Agricultural Policy at a Glance. https://ec.europa.eu/info/food-farming-fisheries/key-policies/common-agricultural-policy/cap-glance_en.
10. Pe'er, G., Zinngrebe, Y., Moreira, F., Sirami, C., Schindler, S., Müller, R., Bontzorlos, V., Clough, D., Bezák, P., Bonn, A., et al. (2019). A greener path for the EU common agricultural policy. Science *365*, 449–451, https://doi.org/10.1126/science.aax3146.
11. Scown, M.W., Brady, M.V., and Nicholas, K.A. (2020). Billions in misspent EU agricultural subsidies could support the Sustainable Development Goals. One Earth *3*, 237–250, https://doi.org/10.1016/j.oneear.2020.07.011.
12. European Union. (2019). Proposal for a REGULATION OF THE EUROPEAN PARLIAMENT AND OF THE COUNCIL establishing rules on support for strategic plans to be drawn up by Member States under the Common agricultural policy (CAP Strategic Plans). vol. 10103/1/19. https://eur-lex.europa.eu/legal-content/EN/TXT/?uri=COM:2018:392:FIN.
13. European Commission (2017). The Future of Food and Farming. https://ec.europa.eu/eip/agriculture/en/news/future-food-and-farming.
14. European Parliament and Council. (2013). REGULATION (EU) No 1306/2013 of the EUROPEAN PARLIAMENT and of the COUNCIL of 17 December 2013 on the Financing, Management and Monitoring of the Common Agricultural Policy and Repealing Council Regulations (EEC) No 352/78, (EC) No 165/94, (EC) No 2799/98, (EC) No 814/2000, (EC) No 1290/2005 and (EC) No 485/2008. https://eur-lex.europa.eu/legal-content/EN/TXT/HTML/?uri=CELEX:32013R1306&from=en.
15. European Commission. Beneficiaries of CAP Funds: Beneficiaries by Country. https://ec.europa.eu/info/food-farming-fisheries/key-policies/common-agricultural-policy/financing-cap/controls-and-transparency/beneficiaries_en.

16. Open Knowledge Foundation Germany. Farmsubsidy.org FAQs. https://farmsubsidy.org/faq/.
17. OpenSpending/Drewes, H. (2014). Farmsubsidy.org Developer Documentation. https://farmsubsidy.readthedocs.io/en/latest/.
18. (2014). Bundesanstalt für Landwirtschaft und Ernährung - Empfänger EU-Agrarfonds – Suche. https://www.agrar-fischerei-zahlungen.de/Suche.
19. Agri, D.G. (2018). Transparency/Working Document Rev6 Ares(2018) 1833750 - 04/04/2018. http://cap-payments.defra.gov.uk/Download/Ares(2018)1833750-rev6-Measures_description-EN.pdf.
20. EAFRD (Pillar II) payments for the programming period 2007-2013 were allowed until December 31, 2015, according to Art. 71 para. 1 VO (EU) Nr. 1698/2005.
21. European Commission (2019). EU Expenditure and Revenue, 2014-2020. https://ec.europa.eu/budget/graphs/revenue_expediture.html.
22. European Parliament (2019). Second Pillar of the CAP: Rural Development Policy, p. 4. http://www.europarl.europa.eu/ftu/pdf/en/FTU_3.2.6.pdf.
23. Andersson, A., Höjgård, S., and Rabinowicz, E. (2017). Evaluation of results and adaptation of EU rural development programmes. Land Use Policy *67*, 298–314, https://doi.org/10.1016/j.landusepol.2017.05.002.
24. Scown, M.W., and Nicholas, K.A.. EU-Agricultural-Systems-Database. https://github.com/murrayscown/EU-Agricultural-Systems-Database.

Patterns, Volume *2*

Supplemental information

A harmonized and spatially explicit dataset from 16 million payments from the European Union's Common Agricultural Policy for 2015

Kimberly A. Nicholas, Frida Villemoes, Edmund Aristid Lehsten, Mark V. Brady, and Murray W. Scown

Supplemental Experimental Procedures

1. Data Sources

1.1. CAP Spending Reporting by EU Member States

The CAP spending data originally came from EU Member States, who are obligated to report spending to comply with the EU's principle of transparency, including financial regulations adopted in 2012 to publish information on recipients of EU funds, and a 2013 regulation with specific obligations for publishing CAP payment recipients.

Specifically, in Article 111 of Regulation (EU) No 1306/2013,[1] Member States are required to report the following information on a single website for at least two years following publication: payment beneficiaries (first and last names of individuals, or full legal name of associations or companies); the municipality where the beneficiary is registered (and postal code "where available"); the amounts of payment corresponding to each measure; and "the nature and description of the measures" for both EU and Member State contributions.

Regarding data accessibility, the European Commission states that *"anyone who receives EU funding under the CAP is included on a publicly available list. This list is designed to promote transparency and trust in EU funding measures. However, the rules still need to strike a balance between the rights of the public to know how their money has been spent and the rights of individuals to protect their personal data. The data is therefore limited in its scope and only available for a set period. [...] It is available from the 31 May of the year after the payments were made. This information will be publicly available for two years after its publication."*[2]

Some CAP beneficiaries are exempted from reporting requirements when the benefits they receive are small, or may be anonymized in some cases. For example, Article 112 of Regulation (EU) No 1306/2013[3] exempts reporting requirements for beneficiaries who receive less than €1250 per year. Additionally, some countries give codes to recipients (although some codes consist of recipient names and locations) or anonymize their data in certain cases. For example, Denmark does not publish the municipality and postal code of anonymous beneficiaries if there are fewer than 10 beneficiaries within a municipality.[4] A few Member States list open data reuse policies on their websites. For example, the UK states that the CAP data are under an Open Government License for public sector information, where users are "encouraged to use and re-use, free to copy, publish, distribute and transmit the information."[5]

The European Commission maintains a webpage[6] with links to each country's CAP payments reporting website, where they state, "*To ensure full transparency, EU countries publish information relating to the beneficiaries of all common agricultural policy (CAP) payments on their national websites."* Each Member State currently maintains its own database for reporting CAP spending, all of which use different formats, with no universal standard for the "nature and description of measures." A few Member States have made it possible in recent years to directly download the full data from current years (e.g., Spain, UK, Hungary, and Portugal), but most national transparency websites only allow searching by specific recipients, measure name, or locations, or using pulldown menus, with results reported in html or displayed as only a few entries at a time (e.g., UK and Sweden). Thus, it is very difficult to get a comprehensive overview of where CAP spending went and for what it was intended.

1.2. Open Knowledge Foundation Germany & Farmsubsidy.org

Because each Member State has their own webpage for reporting CAP spending data, and each uses a different format, obtaining the raw data in a format suitable for analysis presents a significant challenge. We used raw CAP payment data curated by FarmSubsidy.org, which is a project of the Open Knowledge Foundation Germany, a non-profit organization working on transparency of public money. The Farmsubsidy.org project was started in December 2005 by three journalists. Over the years, project

members have worked to “file freedom of information requests on a national level and to clean, compile and present the obtained data on the new Farmsubsidy.org website.”[7] The stated aim of the Farmsubsidy.org project is to “…obtain detailed data relating to payments and recipients of farm subsidies in every EU member state and make this data available in a way that is useful to European citizens.”[8]

The Open Knowledge Foundation works to extract the data currently reported separately by Member States and make the raw data files available in one place. To do so, they have built data scrapers for each country website, which are released under open license with the intention to be maintained by the community.[9] The code for the farmsubsidy.org scrapers are available on GitHub; most were last updated between 2016-2018, although Cyprus was added in late 2019.[10]

Farmsubsidy.org states that they publish the data exactly as published by national governments: “Ultimately, the data available on this site is only as good as the data we have received from the governments. We do not change the data we have received from governments so if you think you have identified an error in the data you should notify the relevant government agency and, if possible, let us know too.”[11]

The Farmsubsidy.org project discloses their funding on their website, which states that most project funding was received between 2006-2010, with the last funding listed as €5,000 in 2014. Their website states “Currently this project is not funded, but maintained by volunteers.”[12]

1.3. Downloading data and data content

We built on the work already undertaken by farmsubsidy.org to use the data they had scraped and made available. We downloaded the raw CAP payment data for all available Member States and years from FarmSubsidy.org on July 15, 2019, using the Linux command
$wget -r https://data.farmsubsidy.org/latest/. These data can be searched on the farmsubsidy.org website by address given or by amount, but not by standardized measure (scheme) name or geolocation, because these features are not reported in the original data.

Each raw data file from farmsubsidy.org listed information potentially including recipient name and address, amount paid, currency, year, and CAP measure under which the payment was made. Not all Member States report all data. Header names were evaluated manually to be sure to include all relevant data (for example, Romania lists measure names (what other countries call “scheme” in their raw data) under the column heading “scheme_2”). See Table S1 for an overview of the content of the raw data files of CAP payments.

2. Data Availability by Country and Year

2.1. Countries and years included for CAP payments

Data on farmsubsidy.org were available for a range of years that varied by country, though most countries had data available for 2014-2017. We built our database using data from 2015; where this was not available, we used the closest available year (2014 for Denmark, and 2016 for Bulgaria and Sweden). Additionally, we present data from 2016 for the Czech Republic, because the 2015 data contained only about 5% of the data volume and 20% of the payment totals than the previous and following year, and was confirmed to be more than five times smaller than the Eurostat payment reported for 2015. Finland had data only for 2004-2013, during the previous CAP period, and was therefore not included. The data for Italy reports only “Total” for each payment rather than listing specific measures, so it was not possible to classify CAP payments in Italy by measure.

During data validation, we identified anomalously low payments for the Czech Republic in 2015 (leading us to realize the 2015 raw data was only about 5% as long as the files before and after, and we decided to use 2016 which had a full dataset). We note our data for Romania are about a third lower than the

payments from Eurostat, but Eurostat data also report 2015 as a year of substantially lower payments to Romania compared with 2014 or 2016, so we elected to keep our 2015 data.

2.2. National vs. EU measures

Although Article 111 of Regulation (EU) No 1306/2013[13] requires Member States to report payment amounts and "the nature and description of the measures" for both EU and Member State contributions, we did not find a consistent system for distinguishing EU and Member State contributions reported in the payment data. For the purposes of this paper we follow the EU terminology used in the legislation and refer to the various payments that Member States are permitted to make under the CAP as *measures* (which elsewhere are referred to more generally as policy instruments or payment schemes depending on the literature).

As a brief explanation for distinguishing European and Member State financing under the CAP, the CAP is divided into two programmes known as "Pillars": Pillar I, entirely financed by the EU[14] through the European Agricultural Guarantee Fund (EAGF), directs the majority of the CAP budget to support direct payments to farmers (71.3% of CAP spending for 2014-2020) and market measures (4.3%).[15] Pillar II finances the remaining 24.4% of CAP spending through the European Agricultural Fund for Rural Development (EAFRD).[16] Pillar II programs support rural development and environmental measures. They are co-financed by EU funds and regional or national funds.[17]

The European Commission states that funding reported under Pillar II includes both EU and national sources: "For funding from the European Agricultural Fund for Rural Development (EAFRD), the amounts published include both the money received from EU funds and from the EU country. This means that the reported amount reflects the total amount of public expenditure on the project."[18]

A few countries listed the funding source in the name of the measures (e.g., Hungary listed National, EAGF, or EAFRD), but most did not. Some reported only EU-funded payments; e.g., Denmark stated "National aid schemes without EU funds are not published".[19]

The farmsubsidy.org data scraped the raw CAP payment data exactly as reported by Member States,[20] which "may also contain non-EU national subsidies which are sometimes in the same database" (Stefan Wehrmeyer, personal communication).

In the absence of any information about national vs. EU funded support in the raw data, we assumed all measures listed were from EU funding, and matched all that we could to the standardized list of EU measures. We classified any measures listed to have national support (either in the measure name, or from information from the reporting agency) with the ID code National, rather than matching to a specific CAP measure (ca. 1% of total payments).

3. Payment values

3.1. Negative payment values

We included all payment values exactly as reported, including negative payment values, which comprised a small proportion of payments in a few countries. The German CAP payment agency Bundesanstalt für Landwirtschaft und Ernährung (Federal Agency for Agriculture and Food) states that positive amounts in the transparency portal refer to payments from the EU budget to beneficiaries, whereas when goods from a public intervention are sold, the price paid by the buyer for the goods appears as a negative amount, representing revenue for the EU budget.[21]

For nearly all countries, negative payments were non-existent or very small (less than 1% of total payments for the year). For instance, for the largest payment recipient, France, negative payments were about 0.4% of total payments in 2015). The only countries to have such payments be more than 2% were

Cyprus (2.7%) and Greece (13%, dominated by one very large negative payment of nearly €200 million). (See file "missing_money_percentages" on our GitHub.) We checked the current data on the Greek transparency website[22] and found 12,024 negative values reported from 2017, so we assume these payments were valid.

3.2. Payments between countries

In the first step of our Python code, payments are summed by country that reported receiving the payment (as the EU reports). But two countries (Belgium and the Netherlands) report payments to other countries within their national transparency reporting, perhaps indicating that the landowner lives abroad. In the second condensation step of our data processing (Figure 1), our Python code attributes all payments to the beneficiary country listed.

For example, payments listed in the raw data reported by Belgium as having a recipient in France would be added to our translated file for France, rather than Belgium. However, these international payments were only reported by Belgium and the Netherlands, and in both cases they were a small percent of the total country payment (about €2.6 million for Belgium, less than half of 1% of their total payments received; and about €13,000 for the Netherlands in 2015, compared with their total payments received of over €1 billion). See the code for "money_movement.py" on our GitHub.

4. Standardizing location and currency

4.1. Location

To obtain a standardized geolocation for each payment entry, we extracted postal codes given in the raw CAP payment data, and matched these to NUTS3 regions, developed and maintained by the EU and generally corresponding to the finest-scale level above municipalities, such as counties or provinces depending on country nomenclature. For example, NUTS3 units in France correspond to 101 Departments.[23] We used the 2013 version of NUTS3, which was appropriate for our 2015 data.[24] We downloaded conversion files from postal codes to NUTS3 regions using the postal code to 2013 NUTS3 regions correspondence table available from Eurostat individually for each country[25] (also found on our GitHub) and used the Python code to extract country and postal codes and match them to NUTS3 regions.

For data where postal codes in the raw data did not match to the available NUTS3 files, we manually matched them where possible by determining the classification system for each country and looking at maps of postal codes and NUTS3 regions to determine the coding pattern between postal codes, which are generally a subset of NUTS3 regions (see Python code, and descriptions in the column "How to match postal codes to NUTS" in Table S1). For most countries, this reduced the amount of payments that could not be geolocated to less than 2% of the total payments. However, Sweden's postal code numbering system[26] does not follow NUTS3 borders,[27] so 19.1% of payments in Sweden could not be linked with a NUTS3 region.

For ten countries (Bulgaria, Czech Republic, Estonia, Greece, Ireland, Lithuania, Luxemburg, Latvia, Romania, and Slovenia), postal codes were not given in the raw data. These countries did include a "recipient location" (usually city) but it was deemed too time-consuming to look up postal codes or otherwise couple them to NUTS3 regions (this could not be automated since shapefiles of postal codes are unfortunately proprietary). Luxembourg, however, consists of only one NUTS3 region, so all payments could be allocated to that NUTS3, leaving nine countries where we could not allocate payments to NUTS3 regions (see Table S1). These nine countries with payments only allocated to the national (NUTS0) level rather than finer NUTS3 spatial level represent about €9 billion in total payments (about 15% of total payments in our 2015 dataset).

4.2. Currency

For countries that did not state a currency of reported payments, we assumed payments were made in Euros (cross-checked with payment totals reported by the EU). Seven countries reported payments in a currency other than the euro. These payments were converted to euros using the average conversion rate for the year of payment. We used currency exchange rates reported by the European Central Bank Euro Reference Exchange[28] to obtain the average value of the given currency for the given year. Values were extracted in (currency) to euro, for January 1 to December 31 for the year of interest. All values are given in euros for the year reported (i.e., they are not converted further to a standardized year). Currency conversion rates are listed in the Python code.

Poland did not list a currency for its payments in the 2015 raw data, but its transparency website stated that payments are shown in PLN, and the total reported payments in the raw data for 2015 totaled 27 billion, whereas the true value was known to be around €6 billion, so we converted the reported payments from Poland to euro assuming they were reported in złoty.

5. Standardizing Payment Measures: Creating the "Rosetta Stone"

We digitized the PDF version of "Description of Measures" into a spreadsheet to consistently align measure names with associated legislation and names in national languages. This spreadsheet formed the basis of the "Rosetta Stone" we used to match measure descriptions across countries.

We added additional classification information compiled during our research to aid with both matching of measure names (our creation of the Rosetta Stone document), as original country scheme names sometimes contained reference to e.g., rural development program measure numbers, and to understand the purpose of each measure for further analysis. (See Table S4 for an overview of the structure of the Rosetta Stone document and the source of our classifications.) After this meta-data, the Rosetta Stone document consists of columns for each country with original scheme name reported, translated scheme name (where relevant), and notes on the matching criteria.

Following DG AGRI, we used the measure identifier (a combination of a Roman numeral and Arabic numeral ID) in the first column of the tables in "Description of Measures" to uniquely identify each of the 102 possible measures for the analysis, which is reproduced in the first column of Table S2. This ID cannot be directly related to legislation, but consists of two or three parts: the short heading for each section in "Description of Measures" (I, II, III, IV/A, V/B, VI/A, VI/B, VI/C and VI/D), and a row number (i.e., 1, 2, 3,…) or sub-table number and row number (i.e., 1.1, 1.2, …), such that III.2 is the ID for the second measure listed in section III and V/B.1.10 is the tenth measure in sub-table one of table V/B. Each measure is linked to the underlying legislation via the columns immediately following the ID: Regulation, Title, Chapter, Section, Article where relevant.

Matching the reported measure name to the master list was straightforward for the 14 countries that used the measure ID to report their measures (as noted in "Includes standard numbers of Measure Descriptions" column in Table S1). However, the remaining 13 countries did not use a standardized identifier for measure names (instead using a wide variety of short descriptions in local languages and/or numerical codes with a variety of meanings). This lack of standardization means it is impossible to use the raw data reported by Member States for cross-country analysis, which is why we undertook the harmonization to make the Rosetta Stone. To take a simple example, different countries reported the following names for a measure, all of which we matched to the first measure (I.1, Single Payment Scheme):

"Single payment scheme - title III"
"I.1 - Aide unique découplée à la surface (DPU)"
"I.1 Καθεστώς ενιαίας ενίσχυσης – τίτλος III (ΕΓΤΕ)"
"I.1"

“Guarantee Fund direct support: I.1, Single payment scheme - title III”

More details about the format and examples for each country reporting their measures are given in Table S1.

For those countries whose measures were not reported according to the measure identifier, we identified unique measure names from national languages and manually matched reported country measure names to the 102 individual measures of DG AGRI using a combination of machine translation, native language speakers, and input from national agriculture experts, as we describe in detail in the next sections.

5.1. Extracting unique measure names

We extracted all unique measure names for each raw country file for analysis in our database (N= 27, all of the EU-28 from 2015, except Finland who reported no data for 2015). We then manually matched measure names from countries to the master list of measures in the Rosetta Stone, using the measure numbers and/or descriptions given as follows.

5.2. Translation and uncertainty assessment

To match measure names used by each country, we created three columns in the Rosetta Stone document for each country: original country scheme name or code (pasted exactly from the raw data files), translated country scheme name (for non-English measure names, this contained the best translation of the original scheme name into English where relevant, preferring native speaker translations over automatic ones), and Notes justifying the basis of the match.

For measure names listed in a language other than English, we first used Google Translate to translate an Excel file containing all measure names from the original language to English, and then manually matched as many measures as possible using the translated names and combination of searching and manual matching with the master measure names.

We distinguished the following classifications for the level of certainty of the match between the national measure name with the master measure list, in order of greatest to least certainty:

Match: all information present, both words and numbers where present, was a strong match to the master list. In other words, both words and numbers matched the master list (if the measure consisted of both words and numbers), words matched the master list (if the measure name consisted only of words) and numbers match (if the measure name consisted only of numbers).

Words but not numbers match: when words from a national measure matched with the master list, but either did not contain numbers present in the master list, or contained numbers that differed from the master list. Where possible to identify, the meaning of the numbers was noted (e.g., when they referred to underlying legislation, article numbers, national measures, or other references).

Unique but imperfect match: the best available match, although some information between the original and master list was inconsistent.

Best of multiple possible matches: more than one match was possible, but enough information was given to support matching with a particular measure.

No match: it was not possible to align the original measure name with a master measure name, because the description given did not fit any of the 102 master measure descriptions from DG AGRI. All non-matches were double-checked against the raw data to ensure they had been imported properly, and that errors (e.g., measure names such as “-” or measure names that cut off in mid-sentence) had indeed been present in the original data and not introduced during our import or analysis.

Note that some measures were placed in both the category "Words but not numbers match" and "Best of multiple possible matches," as they were matched with the best of a number of options, but the numbers did not match.

Note that most countries either had more measure names in their transparency reporting data than the 102 present in the master list (e.g., Hungary listed 274 unique measure names), or listed more than one national measure that was ultimately matched to the same "Description of Measures" name. In such cases, an overflow row was created in the Rosetta Stone, proceeding in alphabetical order by country, with one row per additional measure name. Thus, the first 102 elements of the Rosetta Stone document (rows 2-103, following the header) contain the first instance of a match for an original language scheme name across all countries, with potentially many matches across many countries for each row. Subsequent matches within a country start at row 103 in the Rosetta Stone and continue to row 795, thus there were 692 individual measures added after the main data, where each row lists only one country scheme name, and abbreviated information in the first columns to show the match with the master scheme name.

5.3. Native speaker assistance

For many countries, a substantial fraction of measures remained unmatched at this stage, and we sought the help of native language speakers to improve upon the translations suggested by Google Translate. In many cases native speaker assistance made it possible to make a successful match to align reported measures with the master list.

Native speakers were recruited by the first author on a volunteer basis in an email explaining the purpose of the study, requesting their help in translating into English the measure names that remained unmatched (typically 10-30 short phrases, requiring less than an hour of work), and promising acknowledgement in and a copy of the resulting scientific publication in exchange for their assistance. One native speaker (of Hungarian) was paid as a research assistant for approximately 10 hours of her time in researching Hungarian law as well as providing translations, which helped place many additional measures. We gratefully acknowledge the contributions of all of the native speakers in helping to create this dataset; please see the full list in the Acknowledgements.

5.4. Searching national agency websites

For all countries with measures still not matched to the master list at this stage, the first author searched national agency web pages for any additional information that might assist with matching measure names. In a few cases, it was possible to triangulate information that now appears on national transparency websites to assist in making further matches (e.g., when original language and English translations of measure names were both available), or to download a full list of measure names in their original language with enough information to make a match. These were noted in the relevant country "Notes" column of the Rosetta Stone file.

5.5. Contact with national offices and country experts

After following the procedure above, where any non-matches for measure names remained, the first author sought contact with national agricultural offices in charge of administering CAP payments. Contact information was found from following the links from the official Member States CAP transparency pages, as well as independent research, including seeking contact via the official payment agency Twitter account where available. These details were not always easy to find; to facilitate future contact for others, public email, webpage, and Twitter accounts are listed in Table S1.

The majority of contact attempts with national agricultural agencies to clarify measure names received no reply (contact by email and/or Twitter to the official contact listed for France, Romania, Hungary, Sweden, Lithuania, Bulgaria, and Croatia). Phone contact was successfully made with the UK and Germany, but attempts to follow up with the relevant expert were not successful to receive further information.

(However, contact was successfully made with a CAP expert at an NGO in Germany, who provided additional translations and confirmed matches of measure names to the standard EU list in the Rosetta Stone) (Christian Rehmer, personal communication, 2 March 2020). Twitter contact was successfully made with the agricultural agency in Ireland,[29] but the promised answer from the appropriate section was never received. In several cases, no contact information was readily available (for example, the Austrian CAP payments database website[30] was made by a marketing company; the government agency administering the payment was not clearly listed).

Replies to requests for measure information were received from five countries: Estonia, Latvia, Czech Republic, the Netherlands, and Germany. Representatives from the agricultural agencies in Czech Republic and the Netherlands sent a full list of native measure names aligned with the master list upon request, which enabled complete measure name matching. A representative from Latvia sent a list of measures aligned which enabled matching of all but eight measures (five of which translated as "Action" plus a number). Representatives from the agricultural agency and ministry in Germany provided helpful responses confirming our matches and clarifying the validity of funding periods for different measures. A representative from Estonia sent a match with the 2007-2013 CAP, which was not possible to translate to the current 2014-2020 CAP. Requests to do so did not receive a further reply.

5.6. Expiration and ambiguity of measure names

Complicating the analysis, four of the Regulations underlying the 102 measures expired during the 2014-2020 CAP period, but remained valid for payments through 2015,[31] so Member States in 2015 used a mix of old and new measure terminology in reporting payments. See the correspondence given by EU Regulation between measures in V/B. and IV/A (Table S6).

In Pillar I, Regulation EC 73/2005 (underlying the seven measures related to direct payments in Pillar I for 2007-2013, starting with Roman numeral I) expired in 2013 and was replaced by Regulation 1307/2013 (now with ten measures in Pillar I, starting with Roman numeral II). This means that for example, a country reporting a measure by the name "single area payment scheme" might refer to either I.2 or II.2, both of which use that terminology.

For Pillar II, Regulation EC 1698/2005 (underlying the 46 rural development measures in Pillar II starting with V/B) expired in 2013 and was replaced by the 25 Pillar II measures starting with IV/A associated with Regulation EU 1305/2013. The single measure on information and promotion VI/B.1 (Regulation EC 3/2008) was repealed and replaced by the same measure under Regulation EU 1144/2014 with ID VI/A.1.

Sometimes countries reported a measure using only a short, ambiguous label. For example, three measures relate to "advisory services": IV/A.2, "Advisory services, farm management and farm relief services"; V/B.1.4, "Use of advisory services by farmers and forest holders"; and V/B.1.5, "Setting up of farm management, farm relief and farm advisory services." When countries listed a measure using only a short descriptor such as "Advisory services," it is impossible to say with certainty which measure was indicated.

Finally, one of the two measures making up measure VI/C.1, the POESI measure, expired in 2013, but the measure remained active under the same ID (VI/C.1) with the subsequent regulation.

5.7. Grouping of measures

At the broadest level, we distinguished between measures in Pillar I (the first 27 listed in Table S2) and Pillar II (the last 75 measures), by a column indicating the corresponding funding source for the two Pillars (European Agricultural Guarantee Fund (EAGF) for Pillar I and European Agricultural Fund for Rural Development (EAFRD) for Pillar II) as the local-language version of this acronym was often used in measure names reported by Member States. For further analysis of measure purpose, please see measures identified as income support to farmers and as "environmental payments," which encompasses

all CAP measures which state in the measure wording the intention to principally benefit nature, the environment, climate, or to promote sustainable farming, see Table S2 in Scown et al. (2020).[32]

5.8. Finalizing matches

To finalize the Rosetta Stone was an iterative process involving updating the placement of measure name matches, running the Python code, examining remaining errors and non-matches reported, and repeating until the only remaining non-matches were genuine. Measure names identified as valid non-matches from the translation exercise (where no appropriate match could be found to the 102 measures) were left out of the Rosetta Stone. All other measure names were matched to the most appropriate measure. The Rosetta Stone contains the most current and up-to-date data justifying translations and matches in the Notes column for each country. Some measure names returned as non-matches appeared identical to existing measures already placed, but were added as additional rows in the Rosetta Stone, with measure names pasted exactly as they appeared, in order to obtain a match. Note that all measure names were converted to strings (since there were some measure names that consisted only of numbers) and that spaces and punctuation marks were stripped from the beginning and end of the string to facilitate matching.

6. Existing reporting of data used for validation

Each Member State submits one or more Rural Development Programs (there were a total of 118 Rural Development Programs for the 2014-2020 CAP[33]), which are available as individual PDF files[34] and not in a database format that would more easily support analysis of spending. Some of the countries that receive the most CAP funding have a large number of different rural development programs for different regions (30 in France, 23 in Italy, 19 in Spain, and 15 in Germany),[35] making it very difficult to examine rural development spending by Member State.

The European Network for Rural Development provides spending breakdowns for a subset of measures within Pillar II, but these are difficult to use for validation of one year of data, as they are reported as aggregated over four to seven years in individual PDF reports for Member States where available, for example for individual measures from 2007-2011[36] or for percentage of spending by measure for each Member State for 2014-2020.[37]

Member States tend to report their spending over the full CAP program period, making it difficult to get an independent report of spending for a specific year. For example, the country factsheets list total spending by Member State, including total finances available for the full funding period, but do not distinguish between measures. For example, Sweden's Rural Development Program 2014-2020[38] states that Sweden will use €4.3 billion of public money from 2014-2020 for rural development, of which they state €1.8 billion is from the EU budget and €2.5 billion is national co-funding,[39] but a further breakdown of Pillar II spending is not given.

Financing for rural development consists of co-financed money from the European Agricultural Fund for Rural Development (EAFRD, €99.6 billion for the 2014-2020 multiannual financial framework, averaging €14.2 billion per year over the seven-year spending period) and regional or national public funds varying by region and measure (consisting of €50.9 billion in regional or national co-funding, and a further €10.7 billion of purely national funding, totaling €61.6 billion),[40] or an average of €8.8 billion per year. Thus, on average across the seven years, €23 billion in total is spent annually on rural development, with about two-thirds coming from the EAFRD and one-third from regional or national co-funding.

References

[1] European Parliament and Council (2013). REGULATION (EU) No 1306/2013 OF THE EUROPEAN PARLIAMENT AND OF THE COUNCIL of 17 December 2013 on the financing, management and

monitoring of the common agricultural policy and repealing Council Regulations (EEC) No 352/78, (EC) No 165/94, (EC) No 2799/98, (EC) No 814/2000, (EC) No 1290/2005 and (EC) No 485/2008. https://eur-lex.europa.eu/legal-content/EN/TXT/HTML/?uri=CELEX:32013R1306&from=en

[2] European Commission (Undated). Common Agricultural Policy: Controls and Transparency. https://ec.europa.eu/info/food-farming-fisheries/key-policies/common-agricultural-policy/financing-cap/financial-assurance_en

[3] European Parliament and Council (2013). Regulation (EU) No 1306/2013 of the European Parliament and of the Council of 17 December 2013 on the financing, management and monitoring of the common agricultural policy and repealing Council Regulations (EEC) No 352/78, (EC) No 165/94, (EC) No 2799/98, (EC) No 814/2000, (EC) No 1290/2005 and (EC) No 485/2008. https://eur-lex.europa.eu/legal-content/EN/TXT/HTML/?uri=CELEX:32013R1306&from=en

[4] Miljø-og-Fødevareministeriet Landbrugsstøtte (Undated). See text under “Hvad bliver ikke offentliggjort” (“What will not be published?”) https://lbst.dk/tilskud-selvbetjening/soeg-i-registre/modtagere-af-eu-stoette/landbrugsstoette/#c33575

[5] The National Archives (Undated). Open Government License for public sector information. http://www.nationalarchives.gov.uk/doc/open-government-licence/version/3/

[6] European Commission (Undated). Beneficiaries of CAP funds: Beneficiaries by country. https://ec.europa.eu/info/food-farming-fisheries/key-policies/common-agricultural-policy/financing-cap/controls-and-transparency/beneficiaries_en

[7] Open Knowledge Foundation Germany (Undated). Farmsubsidy.org at a glance. https://farmsubsidy.org/about/

[8] Open Knowledge Foundation Germany (Undated). Farmsubsidy.org FAQs. https://farmsubsidy.org/faq/

[9] OpenSpending/Drewes, H. (2014). Farmsubsidy.org Developer Documentation. https://farmsubsidy.readthedocs.io/en/latest/

[10] Wehrmeyer, S. (Undated). Scrapers for FarmSubsidy data in the member states. https://github.com/openspending/farmsubsidy-scrapers

[11] Open Knowledge Foundation Germany (Undated). Farmsubsidy.org FAQs. https://farmsubsidy.org/faq/

[12] Open Knowledge Foundation Germany (Undated). Farmsubsidy.org FAQs. https://farmsubsidy.org/faq/

[13] European Parliament and Council (2013). Regulation (EU) No 1306/2013 of the European Parliament and of the Council of 17 December 2013 on the financing, management and monitoring of the common agricultural policy and repealing Council Regulations (EEC) No 352/78, (EC) No 165/94, (EC) No 2799/98, (EC) No 814/2000, (EC) No 1290/2005 and (EC) No 485/2008. https://eur-lex.europa.eu/legal-content/EN/TXT/HTML/?uri=CELEX:32013R1306&from=en

[14] European Parliament (2020). Fact sheets on the European Union - Second pillar of the CAP: Rural Development Policy. https://www.europarl.europa.eu/factsheets/en/sheet/110/second-pillar-of-the-cap-rural-development-policy

[15] European Parliament (2020). Financing of the CAP. https://www.europarl.europa.eu/factsheets/en/sheet/106/financing-of-the-cap.

[16] Pe’er, G., et al. (2017). Is the CAP it for Purpose? An Evidence-Based Fitness-Check Assessment. German Centre for Integrative Biodiversity Research (iDiv). https://eeb.org/publications/53/farming/17992/is-the-cap-fit-for-purpose-a-rapid-assessment-of-the-avidence-dr-guy-peer-and-sr-sebastian-lakner-preliminary-summary-of-key-outcomes-june-2017.pdf

[17] European Parliament (2020). Fact sheets on the European Union - Second pillar of the CAP: Rural Development Policy. https://www.europarl.europa.eu/factsheets/en/sheet/110/second-pillar-of-the-cap-rural-development-policy.

[18] European Commission (Undated). Ensuring the correct payment of CAP funds. https://ec.europa.eu/info/food-farming-fisheries/key-policies/common-agricultural-policy/financing-cap/controls-and-transparency

[19] Miljø-og-Fødevareministeriet Landbrugsstøtte (Undated). See text under "Hvad bliver ikke offentliggjort" ("What will not be published?") https://lbst.dk/tilskud-selvbetjening/soeg-i-registre/modtagere-af-eu-stoette/landbrugsstoette/#c33575

[20] Open Knowledge Foundation Germany (Undated). Farmsubsidy.org FAQs. https://farmsubsidy.org/faq/

[21] Bundesanstalt für Landwirtschaft und Ernährung (Undated). Empfänger EU-Agrarfonds – Suche. https://www.agrar-fischerei-zahlungen.de/agrar_suche_hilfe.html

[22] Hellenic Republic Ministry of Reconstruction of Production, Environment, and Energy (2015). CAP Beneficiaries Payments. https://transpay.opekepe.gr/

[23] Wikipedia (Undated). Nomenclature of Territorial Units for Statistics. https://en.wikipedia.org/wiki/Nomenclature_of_Territorial_Units_for_Statistics

[24] Eurostat (Undated). History of NUTS. https://ec.europa.eu/eurostat/web/nuts/history.

[25] Eurostat (Undated). Postcodes and NUTS, https://ec.europa.eu/eurostat/web/nuts/correspondence-tables/postcodes-and-nuts.

[26]GfK GeoMarketing (Undated). 2-digit postcodes Sverige. https://upload.wikimedia.org/wikipedia/commons/e/ee/2_digit_postcode_sweden.png

[27] Eurostat (2018). SVERIGE - NUTS level 3. https://ec.europa.eu/eurostat/documents/345175/7451602/2016-NUTS-3-map-SE.pdf

[28] European Central Bank (Undated). Euro foreign exchange reference rates. https://www.ecb.europa.eu/stats/policy_and_exchange_rates/euro_reference_exchange_rates/html/index.en.html

[29] Nicholas, K.A. (February 24, 2020). https://twitter.com/KA_Nicholas/status/1231969619465900041?s

[30] Transparenzdatenbank EU (Undated). "Information zur Veröffentlichung von Zahlungen im Rahmen der Gemeinsamen Agrarpolitik der EU" https://www.transparenzdatenbank.at/

[31] EAFRD (Pillar II) payments for the programming period 2007-2013 were allowed until December 31, 2015, according to Art. 71 para. 1 VO (EU) Nr. 1698/2005.

[32] Scown, M.W., Brady, M.V., and Nicholas, K.A. (2020). Billions in misspent EU agricultural subsidies could support the Sustainable Development Goals. *One Earth 3*(2): 237-50. 10.1016/j.oneear.2020.07.011.

[33] European Commission (Undated). Rural development programmes 2014-2020. https://ec.europa.eu/info/sites/info/files/food-farming-fisheries/key_policies/documents/rdp-2014-20-list_en.pdf

[34] European Commission, Directorate-General for Communication (Undated). Rural development programmes by country. https://ec.europa.eu/agriculture/rural-development-2014-2020/country-files/

[35] European Commission (Undated). Number of Rural Development Programs per country (total of 118). https://ec.europa.eu/info/sites/info/files/food-farming-fisheries/key_policies/documents/number-of-rdp-per-country-2014-20_en.pdf.

[36] European Network for Rural Development (2013). Progress Snapshot: Measure 323 – Conservation and upgrading of the rural heritage. https://enrd.ec.europa.eu/sites/enrd/files/assets/pdf/measure-information-sheets/2014-06-19/C_Infosheet_323.pdf

[37] European Network for Rural Development (2017). Priority & Focus Area Summaries. https://enrd.ec.europa.eu/policy-in-action/rural-development-policy-figures/priority-focus-area-summaries_en

[38] European Commission (2015). Sweden's Rural Development Programme for 2014-2020 approved by European Commission. https://ec.europa.eu/info/sites/info/files/food-farming-fisheries/key_policies/documents/rdp-sweden-press-summary-26-05-2015_en.pdf

[39] European Commission (2020). Factsheet on 2014-2020 Rural Development Programme for Sweden. https://ec.europa.eu/info/sites/info/files/food-farming-fisheries/key_policies/documents/rdp-factsheet-sweden_en.pdf

[40] European Commission (Undated). Rural development programmes 2014-2020. https://ec.europa.eu/info/sites/info/files/food-farming-fisheries/key_policies/documents/rdp-2014-20-list_en.pdf
